# CRKL mediates EML4-ALK signaling and is a potential therapeutic target for ALK-rearranged lung adenocarcinoma

**DOI:** 10.18632/oncotarget.8638

**Published:** 2016-04-07

**Authors:** Rong An, Yisong Wang, Donna Voeller, Arjan Gower, In-Kyu Kim, Yu-Wen Zhang, Giuseppe Giaccone

**Affiliations:** ^1^ Center for Cancer Research, National Cancer Institute, Bethesda, MD 20892, USA; ^2^ Lombardi Comprehensive Cancer Center, Georgetown University, Washington DC 20007, USA

**Keywords:** EML4-ALK, NSCLC, CRKL, ALK

## Abstract

Anaplastic lymphoma kinase (ALK) gene rearrangements are oncogenic drivers in a small subset of patients with non-small-cell lung cancer (NSCLC). The ALK inhibitors are highly effective in NSCLC patients harboring ALK rearrangements; however, most patients acquire resistance to the therapy following an initial response. Mechanisms of acquired resistance are complex. We used LC-MS/MS-based phosphotyrosine-peptide profiling in the EML4-ALK rearranged H3122 and H2228 cells treated with ALK inhibitors, to identify downstream effectors of ALK. We then used Western blot, siRNA experiments, cell proliferation, viability and migration assays to validate our findings. We identified CRKL as a novel downstream effector of ALK signaling. We demonstrated that CRKL tyrosine phosphorylation was repressed by pharmacological inhibition or small interfering RNA (siRNA) knockdown of ALK in the ALK-rearranged cells. More importantly, CRKL knockdown attenuated their cell proliferation, viability, and migration, but it had no effect on ALK phosphorylation and expression in these cells. Furthermore, CRKL tyrosine phosphorylation was inhibited by dasatinib (an inhibitor of ABL and SRC kinases), which in combination with the ALK inhibitor crizotinib displayed a synergistic inhibitory effect *in vitro*. In conclusion, our study suggests that CRKL is a key downstream effector of ALK, and combined inhibition of ALK and CRKL may represent an effective strategy for treating ALK-rearranged NSCLC patients.

## INTRODUCTION

Lung cancer remains the leading cause of cancer mortality, accounting for over 150,000 deaths in the US each year, 85% of which are non-small cell lung cancer (NSCLC) [1]. Aberrant ALK fusion gene is identified in 3 to 7% of NSCLC cases, mostly in adenocarcinoma, and the most common fusion is with the echinoderm microtubule-associated protein-like 4 (EML4) [2–5]. ALK-rearrangements lead to constitutive, ligand-independent activation of the ALK receptor tyrosine kinase, and consequentially aberrant activation of its downstream signaling pathways such as PI3K-AKT, STATs, and RAS-RAF-MAPK/ERK [6]. Such discovery has led to the successful development of ALK tyrosine kinase inhibitors to treat patients with ALK rearrangements.

ALK inhibitors have superior response rates and progression-free survival in ALK-positive NSCLC patients, and three drugs (crizotinib, ceritinib and very recently alectinib) are currently approved by FDA. Crizotinib demonstrated superior efficacy over chemotherapy in ALK-rearranged NSCLC patients, and ceritinib was also highly active in patients who had disease progression after crizotinib treatment [7–9]. Ongoing clinical trials with other ALK inhibitors including NMS-E628 (renamed RXDX-101) and alectinib (CH5424802) have also produced very promising results so far [10–12]. Nevertheless, toxicity and de novo resistance are significant clinical problems, and not all patients bearing the same ALK fusion protein respond uniformly to these treatments [13, 14]. Moreover, even in patients who initially benefit from ALK-targeted therapy, acquired resistance is inevitable. The lack of long-term benefit results from the emergence of drug-resistant variants due to genetic alteration in ALK itself or activation of other signaling pathways that can bypass ALK inhibition [15, 16]. Further insights into the molecular underpinnings of response and resistance, and the identification of key downstream effectors of ALK signaling could potentially lead to new treatment strategies in ALK-rearranged NSCLC patients.

## RESULTS

**Figure 1:**
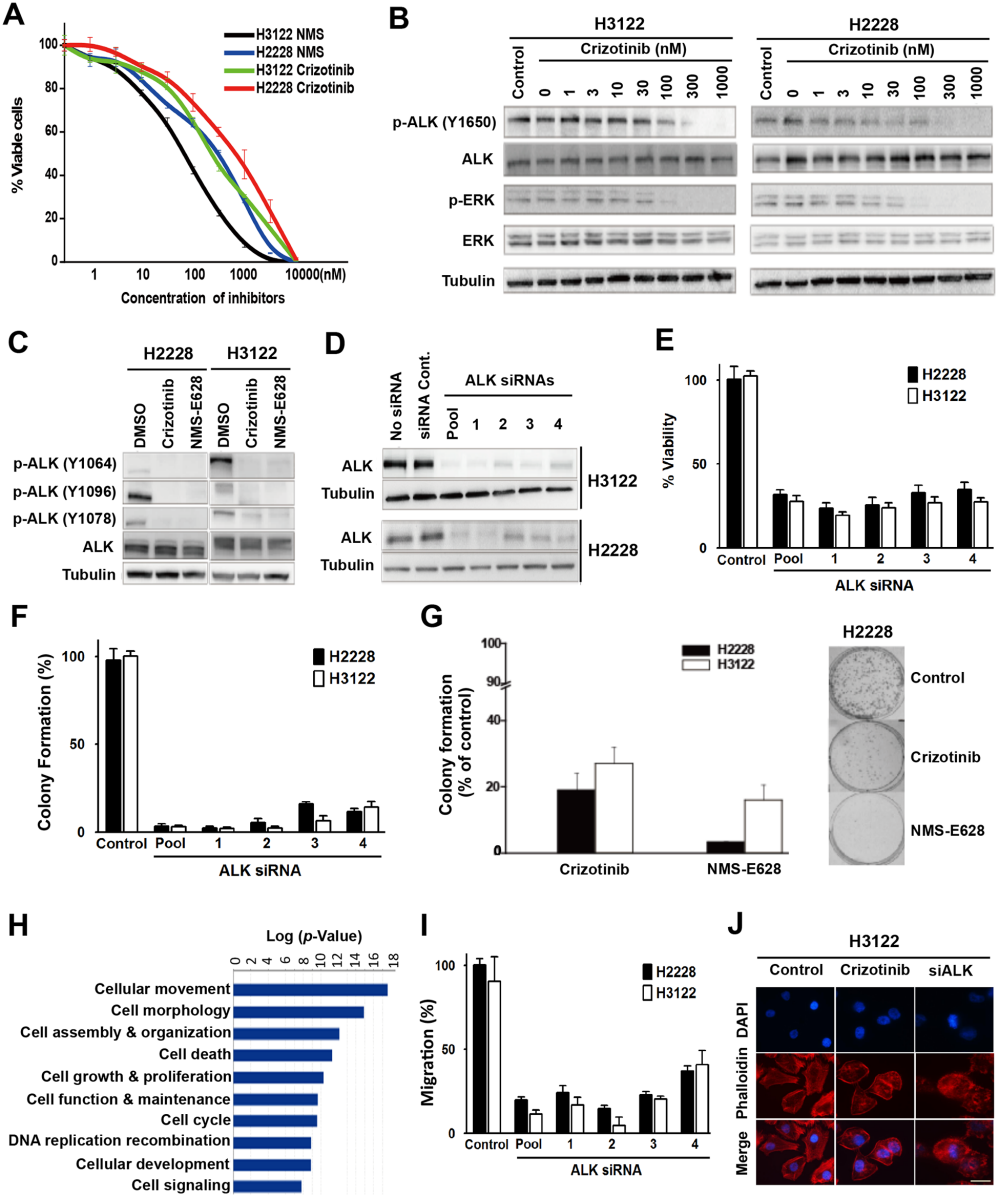
Effects of ALK inhibition in the EML4-ALK-positive NSCLC cells **A.** Cytotoxicity of crizotinib and NMS-E628. H3122 and H2228 cells were grown in 96-well plates and treated with crizotinib or NMS-E628 at the indicated concentrations, and viable cells were measured after 72 hours using an MTS assay. Data are presented as percentage of viable cells in each treatment relative to that in the untreated control cells. **B.** Western blot analyses of ALK and ERK tyrosine phosphorylation in H2228 and H3122 cells treated with crizotinib. Lysates were extracted from cells treated with the indicated drug concentrations, and subjected to Western blotting. Total tubulin was used as a loading control. **C.** Validation of mass spectrometry data of ALK phosphorylation at multiple tyrosine residues. Lysates from H2228 and H3122 cells treated with crizotinib (900nM) or NMS-E628 (300nM) for 1 hr were subjected to Western blotting, probed with three anti-p-ALK antibodies. **D.** Western blot confirmation of ALK knockdown by siRNAs. H3122 and H2228 cells treated with ALK siRNA smartpool of four siRNAs and four individual siRNAs at 20 nM for 72 h. **E.** MTS assay measuring the effect of ALK siRNA knockdown (20 nM for 72 h in triplicate) on the viability of H3122 and H2228 cells (Student's *t*-test: *p*-value < 0.05). **F.** Effect of ALK siRNA knockdown on cell viability measured by colony formation assay. The numbers of foci for each treatment, visualized with Giemsa stain, are the average of duplicate plates from three independent experiments, and are presented as a percentage relative to the number of foci formed in the control plates (Student's *t*-test: *p*-value < 0.05). **G.** Effects of crizotinib (900nM) or NMS-E628 (300nM) on colony formation of H3122 and H2228 cells (Student's *t*-test: *p*-value < 0.05). Right panel shows representative foci of H2228 cells treated with/without crizotinib or NMS-E628. **H.** Ingenuity Pathway Analysis (IPA) of cellular functions reveals high enrichment of proteins involved in cell migration and cytoskeleton modification. The proteins identified by LC-MS/MS with at least 2-fold decrease of phosphorylation after ALK inhibitor treatment were analyzed for association with different cellular functions and with any of 80 canonical pathways and sorted according to their statistical significance of association. The graph represents the analysis of proteins in the crizotinib treated H2228 cells. **I.** Effect of ALK siRNA knockdown on the migration of H3122 and H2228 cell lines treated with ALK siRNA, assessed using quantitative Boyden Chamber technique. **J.** Visualization of F-actin by immunofluorescence staining using anti-phalloidin antibody in H3122 cells treated with/without crizotinib (900nM) or smartpool siALK. Cell images are shown with DAPI in (blue) and phalloidin in (red). Scale bar: 10 μm.

Oncogenic EML4-ALK fusion protein constitutively activates various intracellular signaling pathways [6]. EML4-ALK-positive lung adenocarcinoma cell lines H3122 and H2228 were sensitive to ALK inhibitors crizotinib and NMS-E628 (Figure 1A). The IC_50_ of crizotinib was 300nM for H3122 and 900nM for H2228, whereas NMS-E628 was more potent than crizotinib in both cell lines (Figure 1A). At IC_50_ concentration, crizotinib repressed ALK-dependent phosphorylation of ERK (Figure 1B) and AKT (Figure S1), both being downstream targets of ALK [17]. The IC_50_ concentrations were then used in the subsequent phosphotyrosine peptide profiling experiments to identify tyrosine-phosphorylated proteins in the ALK signaling pathway. This was done by affinity purification of phosphotyrosine peptides, followed by LC-MS/MS identification (Table S1). Proteins showing at least one phosphotyrosine site of greater than 2 folds change between drug-treated and untreated cells for both cell lines were considered significant and analyzed. We identified seven ALK phosphotyrosine sites that were downregulated by both inhibitors (Table S2). Using Western blot analyses, we confirmed the inhibition of three phosphotyrosine sites that are known to be important for NPM-ALK interaction with ALK adaptor proteins [18, 19] (Figure 1C).

We then knocked down ALK with siRNA in H3122 and H2228 cells to determine whether the effects of crizotinib and NMS-E628 on these cells were ALK-dependent (Figure 1D). Similar to crizotinib- and NMS-E628-treated cells (Figure 1A), ALK knockdown resulted in at least 60% reduction of cell viability compared to controls (Figure 1E). Such effects were not observed in the HCC827 and H157 cell lines that carry no ALK rearrangement (Figure S2A). Moreover, both siRNA knockdown and pharmacological inhibition significantly attenuated cell clonogenicity of H3122 and H2228 cells (Figure 1F and 1G), but not that of HCC827 and H157 cells (Figure S2B). These data suggest that crizotinib and NMS-E628 block proliferation and colony formation of H3122 and H2228 cells by inhibiting ALK-dependent activity that is driven by the EML4-ALK fusion oncoprotein.

To explore the biological role of the potential ALK downstream effectors, phosphotyrosine proteins identified by mass spectrometry (Table S3) were subjected to pathway analysis using Ingenuity Pathway Analysis (IPA) algorithm. Cell movement and morphology related pathways were found most significantly enriched after ALK inhibition, with *p* values <10^−17^ and 10^−15^, respectively (Figure 1H). This data suggests that ALK-dependent tyrosine phosphorylation plays important roles in regulating cell morphology and movement. Interestingly, Boyden Chamber assay showed that ALK knockdown significantly inhibited cell migration in the EML4-ALK-positive H2228 and H3122 (Figure 1I), but not in the ALK-wild-type HCC827 and H157 cells (Figure S2C). Moreover, H3122 cells treated with either ALK siRNA or crizotinib were morphologically less elongated or polarized, compared to controls (Figure 1J). These data confirm the IPA results, in that inhibition of EML4-ALK signaling affects cell migration and morphology in addition to other cellular activities such as proliferation and survival (Figure 1E-1H).

**Figure 2:**
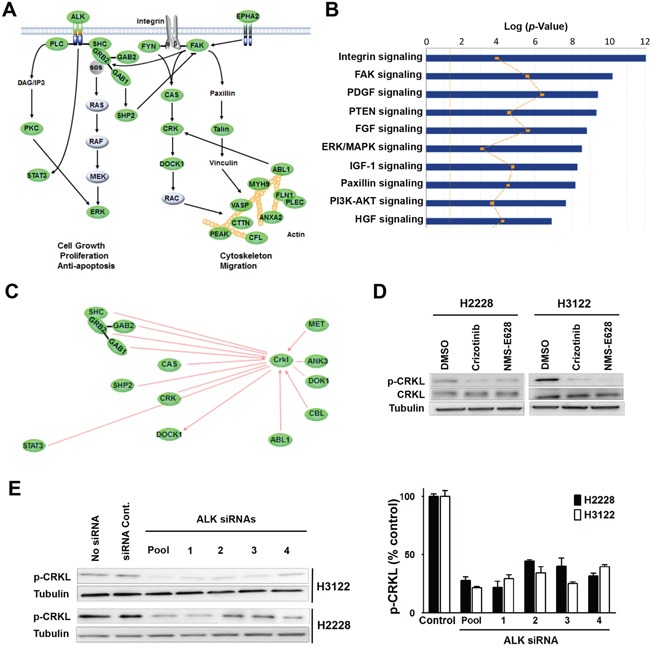
Identification of CRKL as a downstream signaling molecule of EML4-ALK **A.** Regulatory network sensitive to ALK inhibitors in H3122 and H2228 cells revealed by phosphotyrosine peptide mapping. Core signaling proteins inhibited by ALK inhibitors in H3122 and H2228 cells are shown in green. The proteins with ≥2-fold decrease of phosphorylation (at least one tyrosine residue) 1 hr after treatment are presented. **B.** IPA analysis of tyrosine-phosphorylated proteins with differential signaling pathways in H3122 and H2228 cells treated with ALK inhibitor. The yellow line indicates the fraction associated with each pathway of genes that were expressed in each cell line. **C.** Networking of CRKL with various signaling molecules detected in the phosphotyrosine peptide mapping study. Core signaling molecules inhibited by ALK inhibitors in H3122 and H2228 cells are shown in green. **D.** Validation of decreased CRKL phosphorylation identified by mass spectrometry. Lysates from H2228 and H3122 cells treated with/without crizotinib (900nM) or NMS-E628 (300nM) for 1 hr were subjected to Western blot probed with anti-p-CRKL (Y207) antibody. As controls, total CRKL and Tubulin were also detected. **E.** Effect of ALK siRNA knockdown (20 nM for 72 h) on CRKL phosphorylation. Western blot analyses were performed on the lysates from H3122 and H2228 cells treated with ALK siRNAs (four individual siRNA or their smartpool at 20 nM for 72 h) to determine p-CRKL (Y207) level. The graph shows the quantification of p-CRKL levels for each treatment (Student's *t*-test: *p*-value < 0.05).

To further understand ALK signaling in cell proliferation and motility, we examined its downstream and related signal transduction pathways by analyzing the phospho-proteins identified by LC-MS/MS (Table S3). Phosphotyrosine peptide mapping revealed regulatory protein networks of multiple ALK-inhibitor-sensitive pathways in the H2228 and H3122 cells (Figure 2A). These include STAT3, SHC, PLCγ, ERK, and other ALK downstream effectors, which are known to play important roles in cell proliferation, survival, cytoskeleton organization or migration (Figure 2A). Among the proteins whose tyrosine phosphorylation status were repressed by ALK inhibitors, we found significant enrichments of integrin signaling, focal adhesion kinase (FAK) signaling and paxillin/talin signaling (Figure 2B); these pathways are highly related to cell migration and actin cytoskeleton modification. It is noteworthy that those pathways partially overlap with the CAS/CRK/DOCK1 cascade (Figure 2A), which is involved in the regulation of cell motility and morphology [20, 21]. In addition, IPA analysis also identified actin family members and a group of actin binding proteins, including the proto-oncogene ABL1, the myosin protein heavy chain 9 (MYH9), and cortical actin binding protein (CTTN) (Figure 2A).

To identify novel proteins mediating ALK signaling in EML4-ALK positive NSCLC cells, we analyzed the phospho-protein-network before and after ALK inhibitor treatment. Phosphorylation of CRKL (Y207 and Y198), an oncogene known to interact with many molecules linked to the ALK pathway, was significantly decreased in ALK inhibitor treated cells (Figure 2C and Table S3). Western blot analysis confirmed the reduction of CRKL Y207 phosphorylation in H2228 and H3122 cells treated with either crizotinib or NMS-E628 (Figure 2D). To exclude potential off-target effect of ALK inhibitors, we examined the effect of ALK siRNA knockdown on CRKL tyrosine phosphorylation in H3122 and H2228 cells. We found that ALK knockdown also significantly reduced phospho-CRKL level (Figure 2E), indicating that CRKL indeed is a downstream molecule of ALK in the EML4-ALK positive NSCLC cells.

**Figure 3:**
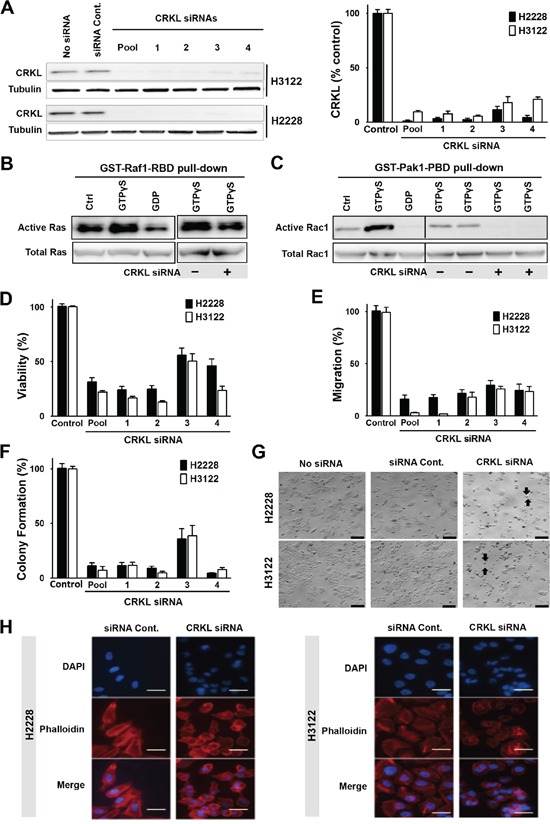
CRKL mediates ALK signaling and regulates cytoskeleton, cell migration and survival **A.** CRKL siRNA knockdown in H2228 and H3122. Protein lysates were extracted from cells treated with four individual CRLK siRNAs (20 nM) or their smartpool for 72 h, and subjected to Western blot analyses. The graph shows the quantification of CRKL proteins. **B.** Inhibition of Ras GTPase activity by CRKL siRNA knockdown. Upper left panel: Lysates of untreated H3122 cells were incubated with buffer (Ctrl), non-hydrolyzable analog of GTP (GTPγS as a positive control) or GDP (as a negative control). Upper right panel: Lysates of H3122 cells treated with or without CRKL siRNA were incubated with GTPγS. Active GTP-bound Ras was pulled down by GST-fusion Ras-binding domain of Raf1 (GST-Raf1-RBD) and detected by immunoblotting with Ras antibody. Lower panels: Total Ras is shown as the input control. **C.** Inhibition of Rac1 GTPase by CRKL siRNA knockdown. Upper left panel: H3122 cell lysates were incubated with buffer (Ctrl), or as positive and negative controls, with GTPγS and GDP, respectively. Upper right panel: H3122 cell lysates with or without CRKL siRNA knockdown were incubated with GTPγS. Active GTP-bound Rac1 was pulled down by GST-fusion p21 binding domain of p21-activated kinase 1 (Pak1) (GST-Pak1-PBD) and detected by immunoblotting with Rac1 antibody. Lower panels: Total Rac1 serves as the input control. **D.** Cell viability assessed 72h after treatment with or without CRKL siRNAs by MTS assay, **E.** Cell migration, assessed using quantitative Boyden Chamber technique (16h after plating), and **F.** Colony formation assays (10 days after plating) were also performed in H3122 and H2228 cells with/without CRKL siRNA knockdown. Data represent the means of at least three independent experiments and are presented as percentage of untreated cells (Student's *t*-test: *p*-value < 0.05). **G.** Bright field images of H2228 and H3122 cells treated with/without CRKL siRNA smartpool or control siRNA. Arrows: round-up cells; Scale bar: 20 μm. **H.** Visualization of F-actin in H2228 (left panel) and H3122 (right panel) treated with either control siRNA or CRKL siRNA smartpool by immunofluorescence staining with phalloidin antibody (red). Blue color indicates DAPI staining. Scale bar: 10 μm.

To determine if CRKL plays a role in mediating EML4-ALK signaling, we knocked down CRKL by siRNAs in H3122 and H2228 cells (Figure 3A). We then examined the effect of CRKL knockdown on the activation of RAS and RAC1, two downstream signaling molecules of ALK. It has been reported that CRKL transforms fibroblasts in a RAS-dependent fashion [22]. We showed that CRKL knockdown attenuated RAS activation in H3122 cells (Figure 3B). Likewise, CRKL siRNA knockdown also significantly inhibited the activation of RAC1, a member of Rho GTPase family, in H3122 cells (Figure 3C). These data indicate that the activation of RAS and RAC1 by EML4-ALK in these cells is in large part mediated by CRKL.

**Figure 4:**
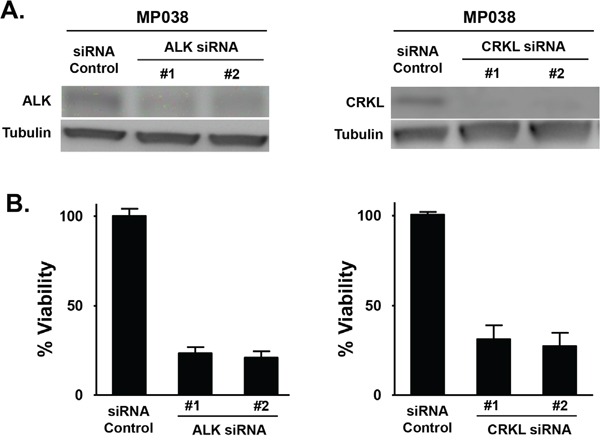
Effect of ALK and CRKL knockdown on ALK-positive MP038 primary NSCLC cells **A.** Western blot analysis showing the knockdown of ALK and CRKL in ALK siRNA- and CRKL siRNA-transfected MP038 cells. **B.** Viability of MP038 cells after transfected with ALK siRNAs or CRKL siRNAs for 72 h. Cell viability (in triplicate) was measured by MTS assay (Student's *t*-test: *p*-value < 0.05).

We further determined the effect of CRKL knockdown on the oncogenic properties of EML4-ALK in these cells. While CRKL knockdown did not affect ALK expression or phosphorylation (data not shown), it significantly reduced cell viability of EML4-ALK positive cell lines but not that of ALK-negative HCC827 and H157 cells (Figure 3D and S2D). Moreover, CRKL depletion reduced cell migration (Figure 3E) as well as colony formation of H3122 and H2228 cells (Figure 3F), but not that of HCC827 and H157 cells (Figure S2E-S2F). Similar to what was observed in the ALK-knockdown cells (Figure 1J), CRKL-knockdown cells also displayed decreased cell adhesion and loss of cell polarity (Figure 3G-3H). In addition, we have successfully established a tumor cell line from an ALK-rearranged NSCLC patient (designated as MP038) as a part of our molecular profiling clinical trial program (Trial ID: *NCI-11-C-0096* and *NCT01306045*) [23]. Knockdown of either ALK or CRKL in this primary tumor line also inhibited cell viability (Figure 4).

**Figure 5:**
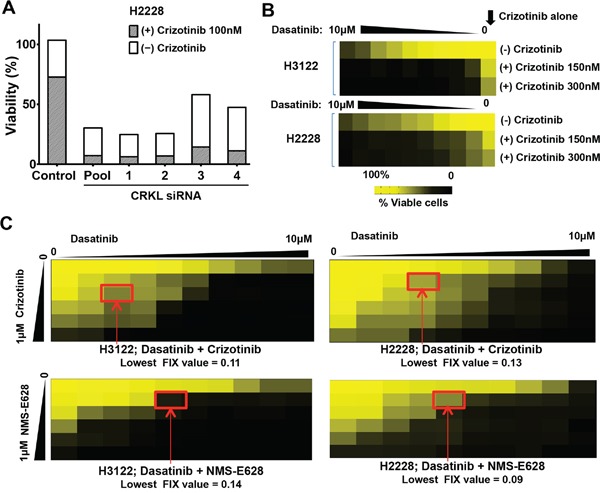
Dasatinib synergizes with ALK inhibitor to inhibit the viability of ALK-positive cells **A.** Cell viability of H2228 cells treated with/without siCRKL siRNAs in the absence and presence of 100nM crizotinib (100 nM), respectively. Data are presented as the percentage of the viable cells with treatment compared to that of untreated cells. **B.** Cell viability (assessed using the MTS assay) of H2228 and H3122 cells treated with crizotinib (150 nM or 300 nM), or dasatinib (from 0 to 10 μM) alone or in combination with 150 nM, or 300 nM of crizotinib. Data are presented as the percentage of viable cells compared with untreated cells. Colored scale bar: percentage of the viable cells with treatment compared to that of untreated cells. **C.** Effects of drug combination of dasatinib and ALK inhibitors (top panels: crizotinib; lower panels: NMS-E628) on H3122 (left two panels) and H2228 (right two panels) cell viability. The dilutions of dasatinib were made horizontally and the dilutions of ALK inhibitors vertically in a 96-well plate. Cells were incubated for 72 h before cell viability was assessed using MTS assay. Data are presented as the percentage of viable cells compared with untreated cells. Colored scale bar: percentage compared with untreated cells. Lowest FIX (Fractional Inhibitory Index) values for each combination in each cell line were calculated according to the Checkerboard Microplate Method (FIX<0.50 is considered synergy).

Given that CRKL mediates ALK signaling and regulates EML4-ALK-dependent biological activities, we hypothesized that inhibition of CRKL sensitizes EML4-ALK-positive cells to ALK inhibitor treatment. We showed that CRKL knockdown drastically enhanced the cytotoxic effect of crizotinib in the EML4-ALK-positive cells (Figure 5A). This data indicates that CRKL is a potential therapeutic target for ALK-rearranged NSCLC, and provides a proof-of-concept for the combination of ALK and CRKL inhibitors as a therapeutic strategy.

Nevertheless, direct pharmacological inhibitors of CRKL are not currently available. We then explored existing compounds that might inhibit CRKL through an indirect drug action mode. It has been previously shown that CRKL is trans-phosphorylated by ABL kinase whereas ABL inhibitors decrease CRKL phosphorylation [24, 25]. We performed MTS assay in H3122 and H2228 cells to determine potential synthetic lethal effect by the combination of crizotinib and ABL inhibitors. A number of structurally unrelated ABL inhibitors including AZD0530 and Bosutinib given at sub-lethal concentrations did not significantly increase the growth inhibitory activity of crizotinib (Figure S3A-S3B) [26, 27]. However, dasatinib, an inhibitor of ABL and SRC kinases, was able to drastically enhance the effect of crizotinib on the EML4-ALK-positive cells (Figure 5B) but not on the ALK-negative cells (Figure S4A-S4B).

We used the Checkerboard Assay Method [28] to measure the effects of drug interactions between dasatinib and ALK inhibitors in the ALK-rearranged cell lines (Figure 5C). Combinatorial effect of the two drugs was determined based on the Fractional Inhibitory Index (FIX) calculated by the method. The lowest FIX values of the dasatinib and ALK inhibitor combination in either cell line were all lower than 0.15 (FIX values <0.5 is considered synergistic). These data indicate that dasatinib and ALK inhibitor combination can achieve a strong synergy in the EML4-ALK-positive cells.

**Figure 6:**
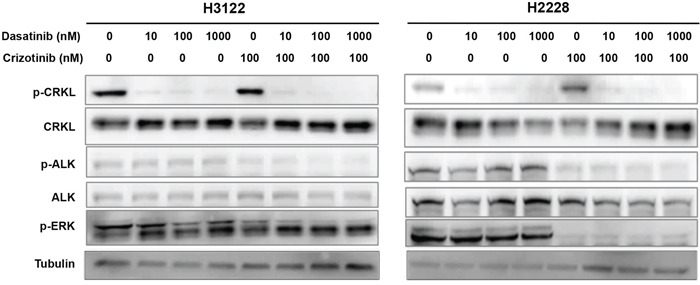
Dasatinib suppresses CRKL activation in the presence and absence of ALK inhibitor H3122 and H2228 cells were treated with dasatinib (0, 10, 100, 1000 nM) and/or crizotinib (100 nM), and cell lysates were prepared 1 hr after the indicated treatment. Western blot analyses were performed to examine the statuses of CRKL, ALK and ERK tyrosine phosphorylation. Tubulin was used as a loading control.

We then performed Western blot analysis to determine the effect of dasatinib on CRKL activation in H3122 and H2228 cells (Figure 6). Dasatinib at 10 nM almost completely abolished CRKL phosphorylation, whereas crizotinib alone at 100 nM had a minimal effect (Figure 6) and required a much higher concentration to inhibit CRKL phosphorylation (Figure 2D). While dasatinib alone had no effect on ALK phosphorylation, its combination with 100 nM of crizotinib (a concentration that is much lower than the IC50 of crizotinib as a single agent) resulted in a strong reduction of both p-CRKL and p-ALK (Figure 6). These data indicate that dasatinib may act through inhibition of CRKL activity to achieve its combinatorial effect with crizotinib in the EML4-ALK positive NSCLC cells.

## DISCUSSION

ALK inhibitors have significant activity in NSCLC harboring ALK fusions [13], however responses are limited in time and patients ultimately acquire resistance within a year of ALK-inhibitor exposure [2]. In-depth understanding of the ALK signal transduction and characterization of factors modulating ALK inhibitor responses may provide crucial information for the development of therapeutic strategies that might improve the efficacy of ALK inhibition and possibly delay or prevent the occurrence of resistance. Acquired resistance to crizotinib can occur through ALK gene amplification, secondary mutations in ALK gene, or via activation of bypass signaling pathways. Such bypass mechanisms include, but are not limited to, KRAS mutations, EGFR activating mutations, SRC activation, KIT amplification, and increased ligand levels activating HER2/3 kinases and EGFR [16, 32, 34, 35]. Crizotinib-resistant patients can be treated with ceritinib, which can overcome several mechanisms of acquired resistance due to crizotinib, including ALK secondary mutations, ALK gene amplifications, and other unknown mechanisms [8, 9]. However, resistance to ceritinib may also develop through mechanisms such as MEK activation and SRC signaling [32]. Other second-generation ALK inhibitors are currently under clinical investigation. A recent phase II global study of ALK inhibitor alectinib treatment in crizotinib-refractory ALK-positive NSCLC showed high response rate, especially in patients with CNS metastases [36], and has recently also been approved by FDA. NMS (renamed RXDX-101 and designated entrectinib) is another well-tolerated second generation ALK inhibitor that is also potent against Trk and ROS1 rearrangements, and is currently under clinical investigation in patients with these genetic alterations (NCT #02097810, NCT #02568267) [37].

In this study, a large-scale proteomics analysis was performed to determine ALK downstream signaling pathways and identified CRKL as a downstream effector of ALK in the EML4-ALK NSCLC cells. CRKL, best known as a substrate of BCR-ABL kinase in chronic myelogenous leukemia (CML) with Philadelphia chromosome, has been described as an oncogene that is amplified in a subset of EGFR-inhibitor resistant NSCLCs [24, 29]. CRKL is predominantly detected in the epithelial cells with low expression in normal lung tissues, and its overexpression is correlated with poor cancer prognosis [25]. Besides BCR-ABL, CRKL has been shown to interact with many other signaling molecules such as GAB1, Paxillin, and STAT5A [30]. In addition, it was reported that C3G in complex with CRKL can bind to the activated ALK [31]. Nonetheless, CRKL as a downstream effector of ALK has never been reported. Here, we characterized the role of CRKL in mediating EML4-ALK signaling and determined the potential therapeutic value of targeting CRKL for ALK-rearranged NSCLCs.

We demonstrated that the activation of CRKL in EML4-ALK-positive cells is ALK-dependent, and is required for the oncogenic activities of EML4-ALK in NSCLC. Knockdown of CRKL blocks the activation of the ALK downstream pathways such as RAS and RAC1, resulting in decrease in survival, motility and transforming activity of these cells. Although with only a limited number of samples (n=4) analyzed, a recent molecular profiling suggests that CRKL amplification does not occur in ALK-rearranged NSCLC patients [32]. This is also true in the EML4-ALK-positive H3122 and H2228 cell lines used in this study, where no CRKL amplification was reported [32]. In the absence of amplification, CRKL can be constitutively activated by aberrant activation of its upstream signaling such as that of EML4-ALK fusion oncoprotein in ALK-rearranged NSCLC. Thus, modulating CRKL activity in ALK-rearranged NSCLC may provide a good opportunity to achieve an enhanced and durable response to ALK inhibitors.

The lack of CRKL specific inhibitors has hindered the exploration of a combination strategy using ALK and CRKL inhibitors. Dasatinib, an approved kinase inhibitor for patients with CML and Philadelphia-positive acute myelogenous leukemia (AML), indirectly inhibits CRKL phosphorylation and synergizes with ALK inhibitors to enhance anticancer activities against ALK-rearranged cells. Due to the multiple other potential targets of dasatinib (e.g. ABL), it is not clear whether inhibition of CRKL by dasatinib is the reason for the synergistic activity. However, it is noteworthy that other ABL inhibitors displayed no activity in combination with crizotinib. In line with our findings, it has been recently reported that SRC signaling mediates acquired resistance to ALK inhibitors and dasatinib sensitizes ALK-rearranged NSCLC to ALK inhibitors [33]. Activation of CRKL by SRC (independent of ALK signaling) is possibly responsible for the resistance. Nevertheless, there is the possibility that mediators other than SRC or ABL could be responsible for the inhibition of CRKL by dasatinib, and the synergy of dasatinib and ALK inhibitor might not be due to CRKL inhibition.

Regardless of what might be accountable for the synergy of dasatinib and ALK inhibitors, CRKL activation is required for ELM4-ALK-dependent signaling and biological activities. Since no direct pharmacological inhibition of CRKL is presently possible, inhibiting CRKL activation indirectly may provide an effective strategy to enhance the efficacy of ALK inhibitors in patients with ALK-rearranged NSCLC.

## MATERIALS AND METHODS

### Cell lines

The H3122, H2228, HCC827, and H157 cell lines were obtained from ATCC, and routinely monitored for authenticity and checked for *Mycoplasma* free using MycoAlert Mycoplasma Detection Kit (Lonza). All these cells were cultured in RPMI 1640 (Sigma) supplemented with 10% fetal bovine serum (FBS), 1% penicillin/streptomycin, and 1 mmol/L sodium pyruvate. MP038 cells were established from pleural effusion of a lung adenocarcinoma patient carrying ALK rearrangement using the conditional reprogramming culture method [23, 34]. This cell line was adapted to grow in RPMI plus 10% FBS in later passages.

### Kinase inhibitors

Crizotinib and dasatinib (BMS-354825) were purchased from Selleck Chemicals. NMS-E628 was from Nerviano Medical Sciences. Stock solutions of all drugs were dissolved in DMSO, aliquots stored at −80°C, and diluted to working concentrations in fresh medium before use.

### Immunofluorescence of F-actin

Cultured cells were fixed in 10% formalin and incubated with the Alexa Fluor 594 phalloidin (Life Technologies) for visualization of F-actin. Nuclei were counterstained with DAPI. The images were taken under an UV-fluorescence microscope.

### Antibodies and western blot analyses

Cells were lysed in buffer containing 50 mM Tris-HCl, pH 7.4, 150 mM NaCl, 0.1% Triton X-100, 5 mM EDTA, 1 mM Na_3_VO_4_, proteinase inhibitors cocktail (Roche) and PhosphoStop phosphatase inhibitor cocktail (Roche). Proteins were separated by electrophoresis on 4-20% polyacrylamide gradient gels, transferred onto membranes using iBlot Transfer Stack (nitrocellulose membrane) with iBlot Transfer System (Invitrogen). Proteins were detected by immunoblotting using an enhanced chemiluminescence system (Perkin-Elmer). Anti-ALK, anti-phospho-ALK (Tyr^1604^), anti-phospho-ALK (Tyr^1096^), anti-phospho-ALK (Tyr^1078^), anti-phospho-CRKL (Tyr^207^), anti-CRKL, anti-ERK1/2 and anti-phospho-ERK1/2 antibodies were obtained from Cell Signaling Technology. The anti-α-tubulin antibody was purchased from Sigma-Aldrich.

### Cell viability assay

Cell viability assay (MTS assay) was performed using the MTS Cell Viability Assay from Promega (Madison, WI) according to the manufacturer's protocol.

### Clonogenicity assay

Cells were grown in RPMI 1640 to 50 - 70% confluence and treated with/without various inhibitors or siRNAs. The cells were then trypsinized, resuspended in the media, counted, reseeded (2,000 per well) into 6-well tissue culture plate, and incubated for 12 days. Fresh medium was added on the 5th day. On the 12th day, medium was removed and the dishes were washed once with ice-cold 1xPBS. The colonies were fixed and stained with 2 mL of 0.25% 1,9-dimethyl-methylene blue in 50% ethanol for 15 minutes. The plates were rinsed three times with 1xPBS and air-dried, and the colonies were counted.

### Migration assay

Migration was assayed using a modified Boyden chamber (Corning Costar, Fisher Scientific, Pittsburgh, PA) containing a polycarbonate membrane filter (6.5 mm diameter, 8 μm pore size). The upper chamber contained cells in RPMI without FBS, and the lower chamber contained RPMI with 10% FBS (chemoattractant) or without FBS (control). Cells were incubated for 16 hours at 37°C in a 5% CO_2_ incubator. Non-migrated cells were scraped off from the upper surface of the membrane with a cotton swab. Migrated cells remaining on the bottom surface were counted after staining with Coomassie Blue.

### Transfection of small interfering RNA

The small interfering RNAs (siRNA) used were ON-TARGET plus SMART pool and 4 individual siRNAs for each targeted molecule obtained from Dharmacon (Chicago, IL). As a control, scramble siRNA was also purchased. Transfection was performed with PepMute™ siRNA Transfection Reagent from SignaGen Laboratories (Gaithersburg, MD) using a reverse transfection procedure as recommended by the manufacturer.

### Phosphopeptide immunoprecipitation

H3222 and H2228 cells (1×10^8^) were treated with DMSO (as control) or crizotinib (10nM, 100nM and 1000nM) for 3hrs. Phosphopeptides were purified by using Cell Signaling PhosphoScan pTyr100 Kits (Beverly, MA) according to the manufacturer's recommendations. Briefly, cells were lysed in urea lysis buffer (20mM HEPES pH 8.0, 9M urea, 1mM sodium orthovanadate, 2.5 mM sodium pyrophosphate, 1mM β-glycerophosphate) at 1x10^7 cells/ml and sonicated. Lysates were cleared by centrifugation at 20,000 x g for 15 minutes, and proteins were reduced by 4.5mM of dithiothreitol and alkylated by 10mM of iodoacetamide. Samples were diluted with 200 mM HEPES pH 8.0 to a final concentration of 2M urea and 20mM HEPES. Trypsin (TPCK-treated; 1mg/ml) was then added to the lysate at 1:100 (v/v) ratio. Samples were digested overnight at room temperature with gentle shaking. Following digestion, lysates were acidified to a final concentration of 1% trifluoroacetic acid (TFA). Peptide purification was carried out using Sep-Pak C18 columns. All elutions (10%, 15%, 20%, 25%, 35% and 40% acetonitrile in 0.1% TFA) were combined into one fraction and lyophilized. Dried peptides were resuspended in 1.4ml of MOPS IP buffer (50mM MOPS pH7.2, 10mM Na_3_PO_4_, 50mM NaCl), and insoluble materials were removed by centrifugation at 2000 x g for 5 minutes. Solubilized peptides were transferred into the microcentrifuge tube containing phosphotyrosine p-Tyr-100 antibody beads (40μl slurry for each sample) and the mixture was incubated overnight at 4°C. The immobilized antibody beads were washed three times with 1ml MOPS IP buffer and twice with 1ml water (ice-cold). Peptides were eluted from beads by incubation with 55 μl of 0.1% TFA, followed by a second elution with 45 μl of 0.1% TFA, and both fractions were combined.

### Analysis by LC-MS/MS

The IP-enriched peptides were analyzed on an LTQ Orbitrap Velos (Thermo Fisher Scientific, San Jose, CA) coupled with a nanoLC system (Eksigent nanoLC-Ultra 1D plus, Dublin, CA). Peptides were separated on a C18 reversed phase column (100mm long, ID 75μm, 5μm 300Å BetaBasic, New Objective, Woburn, MA) using a 90-min linear gradient from 2% to 35% acetonitrile in 0.1% formic acid at a flow rate of 250nL/min. Mass analysis was conducted in data-dependent analysis (DDA) mode, where MS1 scanned mass range from 300 to 2000 m/z at 30,000 resolution in the Orbitrap, and 10 collision-induced dissociation (CID) MS2 scans were sequentially carried out in the ion trap.

The LC-MS data were searched against the SwissProt Human database using Mascot server (Matrix Science, London, UK; version 2.3). Searching parameters were set as: precursor mass tolerance at 20ppm, fragment ion mass tolerance at 0.8 Da, trypsin enzyme with 2 miscleavages, carbamidomethylation of cysteine as fixed modification, and deamidation of asparagine and glutamine, oxidation of methionine, and tyrosine/serine/threonine phosphorylation as variable modifications. Peptides identified from database search were filtered with a false discovery rate (FDR) of 0.05. Relative quantitation of phosphopeptides was calculated based on areas under the curve (AUC) of corresponding peptides from the control and treated samples.

### GTP-RAS and GTP-RAC1 pull-down assay

The pull-down of GTP-bound RAS and RAC1 was performed by the use of active RAS and RAC1 pull-down and detection kits (Millipore) respectively according to the manufacturer's instructions.

## SUPPLEMENTARY FIGURES AND TABLES




