# Supplementary material for: CRKL mediates EML4-ALK signaling and is a potential therapeutic target for ALK-rearranged lung adenocarcinoma
Source: Oncotarget. 2016 Apr 7;7(20):29199–210. doi: 10.18632/oncotarget.8638 (PMC5045389; doi:10.18632/oncotarget.8638)
Supplement: Supplementary file 1 [file oncotarget-07-29199-s001.pdf]

CRKL mediates EML4-ALK signaling and is a potential therapeutic target for ALK-rearranged lung adenocarcinoma

SUPPLEMENTARY FIGURES AND TABLES

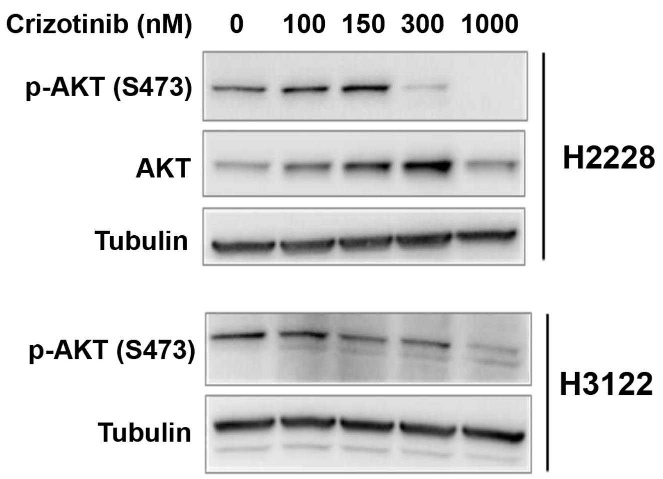

Supplementary Figure S1: Western blot analysis of phospho-AKT Ser473 in H2228 and H3122 cells treated for one hour with Crizotinib at the indicated concentrations. Tubulin was used for loading control.

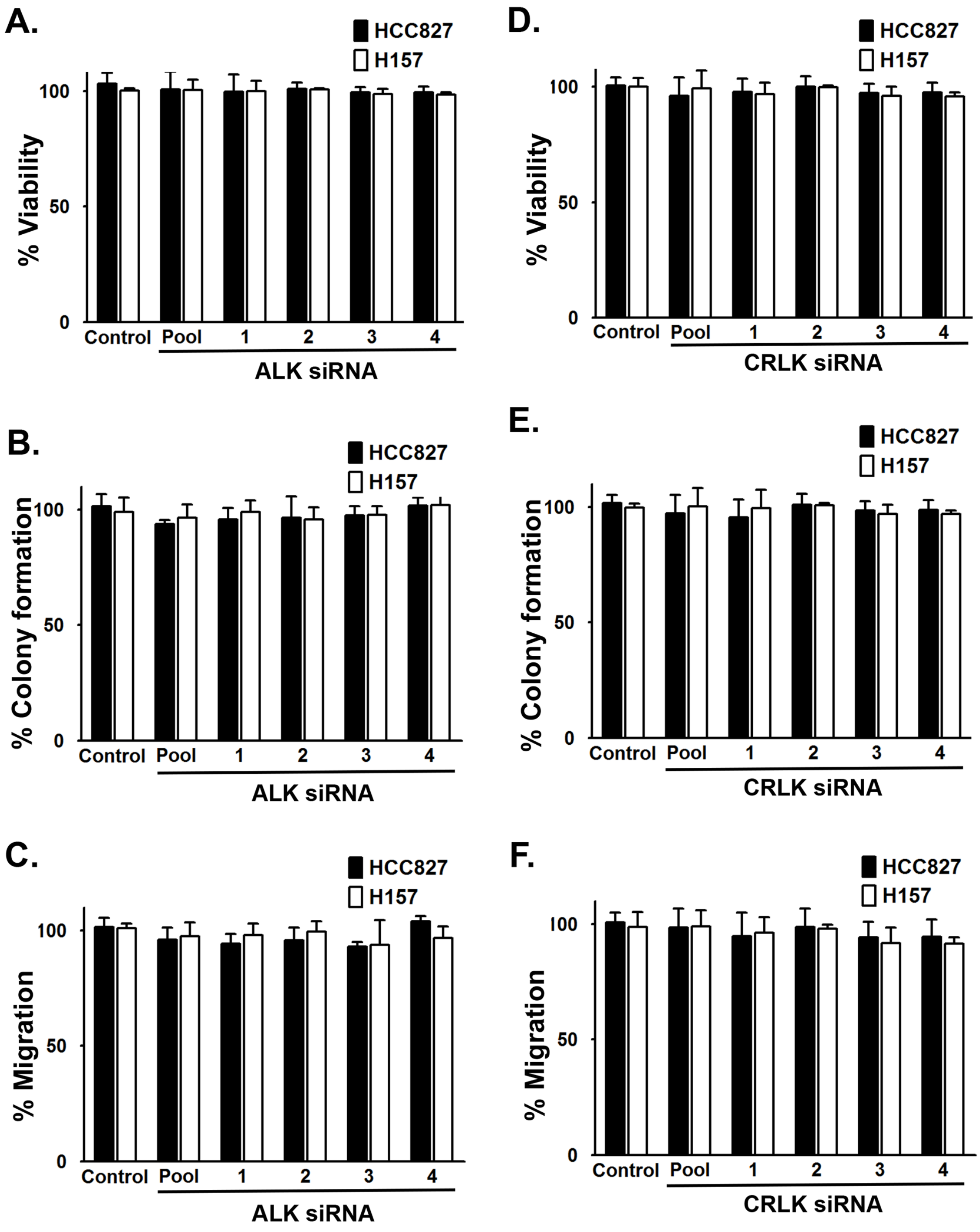

Supplementary Figure S2: Effect of ALK and CRKL knockdown on ALK-negative HCC827, H157 cells. A. Viability assessed using the MTS assay of HCC827 and H157 cells treated with ALK siRNA smartpool of four siRNAs and four individual siRNAs at 20 nM for 72 h. **B.** Colony formation of ALK siRNA-transfected HCC827 and H157 cells 10 days after plating. **C.** Migration assessed using quantitative Boyden Chamber assay of ALK siRNA-transfected HCC827 and H157 cells 16h after plating. **D.** Viability of HCC827 and H157 cells treated with CRKL siRNA smartpool of four siRNAs and four individual siRNAs at 20 nM for 72 h. **E.** Migration assessed using quantitative Boyden Chamber assay of CRKL siRNA-transfected HCC827 and H157 cells 16h after plating. **F.** Colony formation of CRKL siRNA-transfected HCC827 and H157 cells 10 days after plating. All the data represent the means of at least three independent experiments and are presented as the percentage compared with untreated cells.

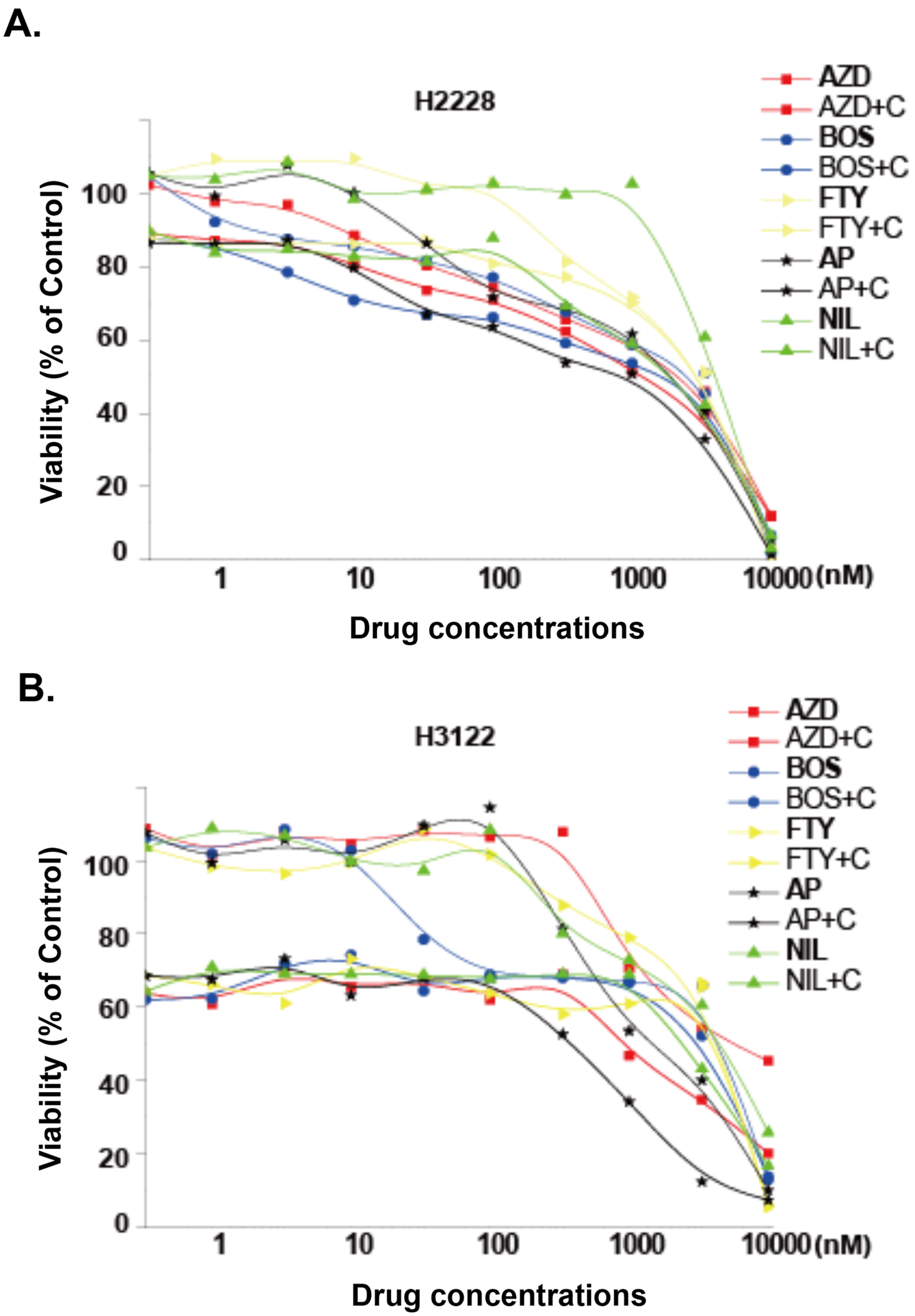

Supplementary Figure S3: Effect of SRC/ABL inhibitors on the viability of ALK-positive NSCLC cell lines. A. MTS analyses of H2228 and **B.** H3122 cells 72 hrs after treatment with the following drugs: AZD (AZD0530); AZD (AZD0530) + C (Crizotinib); BOS (Bosutinib); BOS (Bosutinib) + C (Crizotinib); FTY (FTY720); FTY (FTY720) + C (Crizotinib); AP (ASP3026); AP (ASP3026) + C (Crizotinib); NIL (Nilotinib); NIL (Nilotinib) + C (Crizotinib). AZD0530: a dual inhibitor of SRC and ABL. ASP3026: a second generation ALK inhibitor. Bosutinib: an inhibitor of BCR-ABL and SRC family kinases. Crizotinib: an inhibitor of ALK, ROS1, and MET. FTY720: a sphingosine 1-phosphate (S1P) receptor modulator. Nilotinib: a multi-kinase inhibitor with activities on BCR-ABL, KIT, and several others.

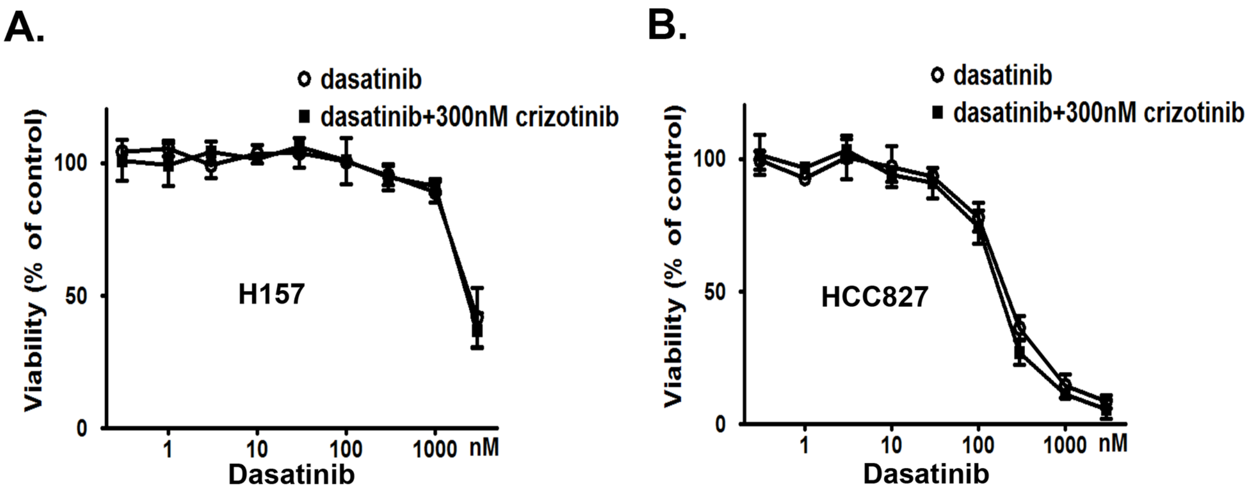

Supplementary Figure S4: Viability of ALK-negative H157 and HCC827 cells treated with dasatinib alone or in combination with Crizotinib (300nM). The viability was measured 72 h after treatment by MTS assay.

Supplementary Table S1: Proteomics overview

Sample ID	Procedure	Number of Peptide	Data Analysis
H3122 (+) DMSO	1) 28-30mg protein/sample;<br>2) Tryptic digestion;<br>3) Peptide purification;<br>4) Lyophilization;<br>5) Immuno-purification with anti-pY100;<br>6) Mass spectrometry	450	1) Peptide de-duplication;<br>2) Unique peptides;<br>3) Peak area intensity;<br>4) Differential regulation of phospho-tyrosine peptides
H3122 (+) Crizotinib		274	
H3122 (+) NMS-E628		311	
H2228 (+) DMSO		568	
H2228 (+) Crizotinib		706	
H2228 (+) NMS-E628		834	

Supplementary Table S2: Phospho-tyrosine residues in ALK identified by LC-MS/MS

Protein	Peptide	pY	PI (DMSO/ Crizotinib)	PI (DMSO/ NMS-E628)	Reported
ALK	HFPCGNVN**Y**^{p}GYQQQGLPLEAATAPGAGHYEDTILK	Y1584	>10	>10	
ALK	HFPCGNVNYG**Y**^{p}QQQGLPLEAATAPGAGHYEDTILK	Y1586	>10	>10	
ALK	HFPCGNVNYGYQQQGLPLEAATAPGAGH**Y**^{p}EDTILK	Y1604	>10	>10	NPM-ALK: PLCγ
ALK	HQELQAMQMELQSPE**Y**^{p}K	Y1078	>10	>10	
ALK	NKPTSLWNPT**Y**^{p}GSWFTEKPTK	Y1507	3.27	5.46	NPM-ALK: SHC
ALK	TSTIMTDYNPN**Y**^{p}CFAGK	Y1096	5.74	7.76	
ALK	TSTIMTD**Y**^{p}NPNYCFAGK	Y1092	5.74	>10	NPM-ALK: IRS1

Supplementary Table S3: The list of phospho-proteins identified by LC-MS/MS with/without ALK inhibitors

2228D/C---ratio of DMSO treated to Crizotinib treated 2228 cells
2228D/N---ratio of DMSO treated to NMS treated 2228 cells
3122D/C---ratio of DMSO treated to Crizotinib treated 3122 cells
3122D/N---ratio of DMSO treated to NMS treated 3122 cells
Red numbers are ALK peptides

See Supplementary File 1
