# Supplementary material for: CRKL mediates EML4-ALK signaling and is a potential therapeutic target for ALK-rearranged lung adenocarcinoma
Source: Oncotarget. 2016 Apr 7;7(20):29199–210. doi: 10.18632/oncotarget.8638 (PMC5045389; doi:10.18632/oncotarget.8638)
Supplement: Supplementary file 2 [file oncotarget-07-29199-s002.pdf]

Sequence	Protein Accessions	2228D/C	2228D/N	3122D/C	3122D/N
MQQNGyENPTYK	A4_HUMAN				
LMTGDTyTAHAGAK	ABL1_HUMAN	1.769647019	1.873190443		
TKPSDEEMLFIyGHYK	ACBP_HUMAN	-10.34603215	0		
KPTyDPVSEDQDPLSSDFKR	ACK1_HUMAN	1.033190098	0.424137546	0.097456646	0.159060475
IWHHTFyNELR	ACTB_HUMAN	0	-6.97440785		
TTGIVMDSGDGVTHTVPIyEGYALPHAILR-19	ACTB_HUMAN	0	-4.939226725	0	-8.09155708
TTGIVMDSGDGVTHTVPIYEGyALPHAILR-22	ACTB_HUMAN			0	-8.09155708
DSyVGDEAQSK	ACTB_HUMAN	1.376656283	0.259204352		
GySFTTTAER	ACTB_HUMAN	0.053972798	-0.990467606		
AIMTYVSSFyHAFSGAQK	ACTN1_HUMAN;ACTN4_HUMAN	-2.678952579	4.579097631		
mVDyHAANqSyQyGPSSAGNGAGGGSMGDYMAQEDDWDR	ACTN4_HUMAN			6.846050555	6.846050555
FAVPTyAAK	ADAM9_HUMAN	0.740107603	1.076140299		
VSSQGNLIPARPAPAPPLySSLT	ADAM9_HUMAN	0.48432629	9.095593015		
VAQQPLSLVGcEVVPDPSPDHLySFR	AF1L2_HUMAN	-8.496974779	-8.638194423	0	-8.763457316
AGLQTADKyAALANLDNIFSAGQGGDQGSGFGTTGK	AGFG1_HUMAN	0	-5.595511073		
VKGEyDMTVPK	AHNK_HUMAN	0.694455386	2.148460798		
VKGEyDVTVPK	AHNK_HUMAN	-0.123519273	0.401940077		
GPNVKGEyDVTMPK	AHNK_HUMAN	1.482713302	7.428574533		
MKGDyDVTVPK	AHNK_HUMAN	0.587932545	2.097752678		
VTAyTVDVTGR	AHNK_HUMAN	0	-4.586789602		
LNDGHFMPVLGFGTyAPAEVPK	AK1C1_HUMAN;AK1C2_HUMAN	0.744368868	-0.605223231		
HIDSAHLyNNEEQVGLAIR	AK1C1_HUMAN;AK1C3_HUMAN	-7.394969431	-8.74825075		
LNDGHFMPVLGFGTyAPPEVPR	AK1C3_HUMAN	0	-6.986669249		
NLHYFNSDSFASHPNYPYSDEy	AK1C3_HUMAN	-7.966375632	0		
PyQYPALTPEQK	ALDOA_HUMAN	-5.608461045	-5.950487392		
NKPTSLWNPTyGSWFTEKPTK	ALK_HUMAN	3.736755446	2.232889108	1.707838406	2.449508588
NKPTSLWNPTyGSWFTEKPTKK	ALK_HUMAN	2.77551749	1.379935298	0.996941348	1.759910505
TSTIMTDYNPNycFAGK-12	ALK_HUMAN	10.80946452	10.80946452	2.521014343	2.95567563
TSTIMTDyNPNYcFAGK-8	ALK_HUMAN			2.521014343	2.95567563
HQELQAMQMELQSPEyK	ALK_HUMAN	9.718207375	9.718207375	8.895450456	8.895450456
KHQELQAMQMELQSPEyK	ALK_HUMAN	9.331519066	9.331519066	9.026528314	9.026528314
HFPcGnVNYGyQQQGLPLEAATAPGAGHyEDTILK-11	ALK_HUMAN			9.586576505	9.586576505
HFPcGnVNyGYQQQGLPLEAATAPGAGHyEDTILK-9	ALK_HUMAN			9.586576505	9.586576505
HFPcGNVNYGYQQQGLPLEAATAPGAGHyEDTILK-29	ALK_HUMAN	8.580682424	8.580682424		
ADIVQQLLQQGASPNAATTSGyTPLHLSAR	ANK3_HUMAN			7.47315667	7.47315667
GGPGSAVSPyPTFNPSSDVAALHK	ANXA1_HUMAN	0.450806751	0.256140966		
ALyEAGER	ANXA1_HUMAN	0.072538195	-0.4480718		
ALyEAGERR	ANXA1_HUMAN	-0.340097601	-0.934084678		
SLYyYIQQDTK	ANXA2_HUMAN	0	-6.450266198		
SYSPyDMLESIRK	ANXA2_HUMAN	-1.252083826	0.801281541		
LyDSMK	ANXA2_HUMAN	0	-5.900579922		
AyTNFDAER	ANXA2_HUMAN	0.801691217	0.587830734	1.105018587	0.77784689
LSLEGDHSTPPSAyGSVK	ANXA2_HUMAN	-0.634967671	-0.512507984	2.84495484	1.041056187

SYSPyDMLESIR	ANXA2_HUMAN	0	-7.320485441		
DLyDAGVKR	ANXA2_HUMAN	-6.44734947	-6.938119354		
LYDAyELK	ANXA5_HUMAN	-5.474526696	-6.516033521		
MQNHGYENPTyK-11	APLP2_HUMAN	-0.824040716	3.386573336		
MQNHGyENPTYK-6	APLP2_HUMAN	-1.178764021	4.253635378		
RTEELIyLSQK	ARHG5_HUMAN	-1.510075274	-1.713474721	-0.189472809	-0.453089379
PLyEGLGSGGEK	ARP10_HUMAN	-6.330630775	-5.769738882		
GIVVyTGDR	AT1A1_HUMAN	-8.961739578	-7.489772061	0.586281652	0.349152497
DKyEPAAVSEQGDKK	AT1A1_HUMAN	-6.560073795	-6.694459854		
LSyRHGLLGVDK	ATG2B_HUMAN	6.615851173	6.615851173		
AQQGLyQVPGPSPQFQSPPAK	BCAR1_HUMAN	1.235740443	1.278253281	-0.16814911	-0.091988951
HLLAPGPQDIyDVPPVR	BCAR1_HUMAN	-0.236474778	-1.955606655	-0.400188881	0.177336901
RPGPGTLyDVPR	BCAR1_HUMAN			7.917623718	-0.252615254
VLPPEVADGGVVDSGVyAVPPPAER	BCAR1_HUMAN	0	-6.478761607		
LSEAySR	LSEAySR	LSE LSEAySR	LSEAySR	LSEAySR	LSEAySR
cLEEHyGTSPGQAR	BCAR3_HUMAN	0	-5.742534055		
TVMYTAVGSEWRPFGyPR	BCS1_HUMAN	-1.251330023	-1.868427312		
VFDKDGNGyISAAELR	CALM_HUMAN	0.666333958	1.294405531		
YVDSEGHLyTVPIR	CAV1_HUMAN	0.567590378	1.176201793		
EQGNIyKPNNK	CAV1_HUMAN	-8.473936908	0		
IKPSSSANAIySLAARPLPVPK	CBL_HUMAN	11.69469795	5.726702991		
SLLSHEFQDETDTEEETLySSKH	CBPD_HUMAN	-7.631379062	-7.270591144		
TNKGPAVGIyNDNINTEMPR	CDCP1_HUMAN				
SPPESESEPyTFSHPNNGDVSSK	CDCP1_HUMAN	1.506840039	0.759879314		
GPAVGIyNDNINTEMPR	CDCP1_HUMAN				
IGEGTyGVVYK-6	CDK1_HUMAN	1.319129432	0.891555043	-0.018618868	-0.326988348
IGEGTYGVVyK-10	CDK1_HUMAN			-2.811000004	-3.119369484
IGEGTyGTVFK	CDK5_HUMAN	0.59264303	0.515163657	-8.341233898	-8.496052372
HVHLENATEyATLR	CK052_HUMAN	0.469025214	1.119328747		
ALEHFTDLyDIKR	CLH1_HUMAN	0	-7.306001419		
HELQANcyEEVKDR	COF1_HUMAN	0.615951219	10.2412395		
EVyFAER	CRIP1_HUMAN	0	-6.055036587		
QEEAEyVR	CRK_HUMAN	6.765837039	2.279918546		
NSNSyGIPEPAHAYAQPQTTTPLPAVSGSPGAAITPLPSTQNGPVF	CRKL_HUMAN			3.602318091	0.401229354
VPcAyDK	CRKL_HUMAN				
TLyDFPGNDAEDLPFK	CRKL_HUMAN				
NSNSYGIPEPAHAyAQPQTTTPLPAVSGSPGAAITPLPSTQNGPVF	CRKL_HUMAN	0.108374036	0.148646933	0.474049787	0.401229354
RVPcAyDK	CRKL_HUMAN	-9.561175583	-10.65137113		
NTyNQTALDIVNQFTTSQASR	CSKI2_HUMAN	0	-5.767405931		
LVyDGIR	CTNA1_HUMAN			7.106097801	7.106097801
NAGNEQDLGIQyK	CTNA1_HUMAN	0.211652796	0.645530953		
SLDNNySTPNER	CTND1_HUMAN	-0.308013285	0.335454299	5.916784998	5.916784998
HYEDGYPGGSDNyGSLSR	CTND1_HUMAN	0.629803203	1.064399913		
LNGPQDHSHLLySTIPR	CTND1_HUMAN	-11.52583226	-10.28860168		
IcEVRPcGQPVySSLKK	CYR61_HUMAN	-8.788099573	0		

LEAVqYKTqVVAGTnYyIK	CYTA_HUMAN	-4.047238041	0		
FQDVGPQAPVGSVyQK	DBNL_HUMAN	-0.958099939	-0.962153252		
AGKPGLPAPDELVyQVPQSTQEVSGAGR	DCBD2_HUMAN	-0.195556018	0.497751538		
TEGTyDLPYWDR	DCBD2_HUMAN	-4.716210538	0		
ATGNQPPPLVGTyNTLLSR	DCBD2_HUMAN	-7.237238953	0		
SDyDGIGSR	DDX3X_HUMAN	0.149526354	0.23023242		
GRSDyDGIGSR	DDX3X_HUMAN	0	-4.992461701		
DKDAySSFGSR	DDX3X_HUMAN	-5.899496644	-6.454610203		
YMEySTKK	DGKA_HUMAN				
NLNHVSyGR	DHE3_HUMAN	0	-4.697243698		
GSVADyGNLmENQDLLGSPTPPPPPPHqR	DOCK1_HUMAN	0	-6.747619894	5.629282753	5.629282753
SHNSALySQVQK	DOK1_HUMAN	10.2983108	5.250049577		
EIySAPTGDR	DUOX1_HUMAN	10.91488888	10.91488888		
NYMSNPSYNyEIVNR	DYHC1_HUMAN	-0.643950398	-0.903538371		
IYQyIQSR	DYR1A_HUMAN	0.511575212	0.176733356	0.298592837	0.093280182
KVYNDGYDDDNyDYIVK	DYR1A_HUMAN	0.875409168	0.441474242		
VYNDGYDDDNyDYIVK	DYR1A_HUMAN	-0.871785344	-1.676721118		
LYTyIQSR	DYRK3_HUMAN	-8.829089363	-9.316814032		
VEGDNIyVR	E41L2_HUMAN	0	-4.709218316		
STTTGHLIyK	EF1A1_HUMAN	0.63430881	0.362897964	0.185823767	0.01730529
EHALLAyTLGVK	EF1A1_HUMAN	-0.758404161	-0.94525389	-0.094244972	-0.528275367
TADSVFcPHyEK	EFNB2_HUMAN	-3.390730659	0		
RPAGSVQNPVyHNQPLNPAPSR	EGFR_HUMAN	0.510455367	0.925533639	0.457025955	-0.305279497
GSTAENAEyLR	EGFR_HUMAN	0.24048378	0.379318205	0.523284298	-0.098062154
GSHQISLDNPDyQQDFFPK	EGFR_HUMAN	-0.400409394	-0.200263233	-0.044604956	-0.909416963
DPHYQDPHSTAVGNPEyLNTVQPTcVNSTFDSPAHWAQK-17	EGFR_HUMAN	2.275474202	0.561119167		
LLGAEEKEyHAEGGKVPIK	EGFR_HUMAN	0.983091738	8.158483052		
DPHyQDPHSTAVGNPEYLNTVQPTcVNSTFDSPAHWAQK-4	EGFR_HUMAN	-0.86573845	4.202247528		
MHLPSPTDSNFyR	EGFR_HUMAN	-7.018120363	-7.33095378		
NSESKPKEPVFSAEEGyVK	EMAL1_HUMAN	0.166550546	-0.168546914		
EPVFSAEEGyVK	EMAL1_HUMAN	-10.10015658	-10.35709383		
DVIINQEGEyIK	EMAL4_HUMAN	0.549220099	1.093306423	0.303170287	0.093820582
TADKHKDVIINQEGEyIK	EMAL4_HUMAN	0.503382724	-0.238163423	-0.245737483	-0.336695342
HKDVIINQEGEyIK	EMAL4_HUMAN	0.787139624	0.605124869	0.402890325	0.112335417
LEWAyGYR	EMAL4_HUMAN			9.322268296	9.322268296
LKLEWAyGYR	EMAL4_HUMAN			9.758266058	9.758266058
AAVPSGASTGIyEALELR	ENOA_HUMAN	-2.63939567	-3.692084832		
SEQLKPLKTyVDPHTYEDPNQAVLK	EPHA2_HUMAN	0.540110962	4.015757496		
TYVDPHTyEDPNQAVLK	EPHA2_HUMAN	1.213096975	-0.547988027	3.453703069	3.453703069
VLEDDPEATyTTSGGK	EPHA2_HUMAN	0.724349882	0.493649536	0.787922331	0.499146043
QSPEDVyFSK	EPHA2_HUMAN	1.144468306	0.538100972	8.283986718	8.283986718
ADPPyTHTIQK	EPS8_HUMAN			0	-5.881402662
VySQITVQK	EPS8_HUMAN	6.546622024	6.546622024		
VSSTHyYLLPERPPYLDK	ERRFI_HUMAN	1.432386853	7.149932366		
VSSTHyYLLPERPPYLDKYEK	ERRFI_HUMAN	-0.283498697	0.601813436		

APDFVFyAPR	EZRI_HUMAN				
SELPyEELWLEEGKPSHQPLTR	FA59A_HUMAN	2.579425607	12.02084807	1.210165646	1.15086253
GSIDREDGSLQGPIGNQHIyQPVGKPDPAAPPK	FAK1_HUMAN	-0.459487799	-0.343099295	-0.485166176	-0.402860776
YMEDSTYyK-8	FAK1_HUMAN	2.864995461	2.530150507	0.171938297	0.151005302
YMEDSTyYK-7	FAK1_HUMAN	-0.707278529	-1.317803616	0.171938297	0.151005302
YMEDSTyyKASK	FAK1_HUMAN	0	-3.326144066	-7.317539635	0
THAVSVSETDDyAEIIDEEDTYTMPSTR	FAK1_HUMAN	-8.652137924	-10.60381244		
AFDTAGNGycR	FAS_HUMAN				
VQENDGKEPPPVVNyEEDAR	FER_HUMAN	-0.054110389	0.223832162		
QEDGGVySSSGLK	FER_HUMAN	0.71890034	0.83599457		
KDGyVLR	FETUB_HUMAN	0	-7.998712156		
VHSPSGALEEcyVTEIDQDKYAVR	FLNA_HUMAN			7.526476938	-0.79366595
SSTETcySAIPK	FLNB_HUMAN	0.393490849	0.399623835		
HGHyFVALFDYQAR	FRK_HUMAN			-0.030645208	0.212072387
LVyENINGLSIPSASGVR	FRS2_HUMAN	7.849350539	7.849350539	7.846267777	7.846267777
KLDNGGyYITTR-7	FYN_HUMAN				
LDNGGyYITTR-6	FYN_HUMAN				
LDNGGYyITTR-7	FYN_HUMAN				
LIEDNEyTAR	FYN_HUMAN	1.688199814	1.65305536	1.243888882	1.696521778
GAySLSIR	FYN_HUMAN	0	-6.595632981		
KLDNGGYyITTR-8	FYN_HUMAN	0	-6.889497184		
VGFQyEGTYK-5	G6PD_HUMAN	-0.515101208	-0.961904173		
VQPNEAVyTK	G6PD_HUMAN	-12.39628534	-12.83914904	-8.958533617	-9.262663631
VGFQYEGTyK-9	G6PD_HUMAN	0.941127037	0.385718631		
RVGFQYEGTyK-10	G6PD_HUMAN	0.612403691	0.181563376		
NSYVAGQyDDAASYQR	G6PD_HUMAN	0.504150877	-0.033056394		
RVGFQyEGTYK-6	G6PD_HUMAN	-0.355508875	-0.786349191		
SSGSGSSVADERVDyVVVDQQK	GAB1_HUMAN	9.588829201	9.588829201		
VDyVVVDQQK	GAB1_HUMAN	10.2422608	10.2422608		
DASSQDcyDIPR	GAB1_HUMAN	7.886910134	7.886910134		
AGDNSQSVyIPMSPGAHHFDSLGYPSTTLPVHR	GAB2_HUMAN	1.045268313	9.798722699		
TDDYLDQPcyETINR	GDIB_HUMAN	-7.032245961	-7.40361297		
LATELyHQK	GFPT1_HUMAN	0	-6.378745614		
NTDYTELHQQNTDLIyQTGPK	GOGA5_HUMAN	0	-7.610394595		
VPSEGAyDIILPR	GPC5C_HUMAN	-0.080330504	1.561316753	1.039492285	0.296997079
AEDMySAQSHQAATPPK	GPC5C_HUMAN	5.847348245	-0.696304304	6.507593202	0.627975896
AEDMySAQSHQAATPPKDGK	GPC5C_HUMAN	0.150096665	0.501769901	6.515646586	0.438319441
GVGyETILK	GPC5C_HUMAN	-6.828866009	-6.239019784		
ALyNIGNVYHAK	GPSM1_HUMAN	8.51904736	1.107347573		
NEEENIySVPHDSTQGK	GRLF1_HUMAN	0.676278534	0.685810353	0.70819401	0.449956568
SVSSSPWLPQDGFDPSDyAEPMDAVVKPR	GRLF1_HUMAN	-8.342862899	-6.804586068	-8.661985676	-8.924104619
TymMmFR	GRM1B_HUMAN	-3.189310631	0		
SRGDNVyVVTEVLQTQK	GSDMD_HUMAN			8.282774741	8.282774741
GEPNVSyIcSR	GSK3B_HUMAN	0.428988189	0.047935746	0.05840588	-0.308237327
QEPSEyQDK	HFM1_HUMAN	7.760078533	7.760078533		

AVcSTyLQSR	HIPK2_HUMAN	0.520055517	0.085394567	0.139027907	-0.02254731
TVcSTyLQSR	HIPK3_HUMAN	10.39592486	0.352056036	-0.018309695	-0.180198707
DGMDNQGGyGSVGR	HNRH3_HUMAN	7.692690893	4.843191648		
FMSVQRPGPyDRPGTAR	HNRPF_HUMAN	-7.72181523	0		
GGNRFEPyANPTKR	HNRPM_HUMAN	0	-6.036809907		
LVTSPccIVTSTyGWTANMER	HS90A_HUMAN	0	-7.488715206		
HIyYITGETK	HS90A_HUMAN				
SIyYITGESK	HS90B_HUMAN				
LVSSPccIVTSTyGWTANMER	HS90B_HUMAN	2.349730628	-0.310259885		
LKKEDIyAVEIVGGATR	HSP74_HUMAN	0	-6.893865857		
AySSFGGGR	IF4H_HUMAN	0	-3.562586884		
KGSGDyMPMSPK	IRS1_HUMAN	3.934561092	3.69235061		
LEYyENEKK-4	IRS1_HUMAN	0.691941326	0.454704826	-6.145589067	0
LEyYENEKK-3	IRS1_HUMAN	0.791074258	0.332596065	2.091097814	0.187045598
LEyYENEK-3	IRS1_HUMAN	1.073340921	0.672963218	6.699797277	0.579958383
LEYyENEK-4	IRS1_HUMAN			6.699797277	0.579958383
EEETGTEEyMK	IRS1_HUMAN	9.15033827	9.15033827		
GDyMTMQMScPR	IRS1_HUMAN	6.710062428	6.710062428		
GGHHRPDSSTLHTDDGyMPMSPGVAPVPSGR	IRS1_HUMAN	-0.176817067	10.11900733		
QRPVPQPSSASLDEyTLMR	IRS2_HUMAN	8.944804871	8.944804871		
SDDyMPMSPASVSAPK	IRS2_HUMAN	5.015117193	1.456468818		
APYTcGGDSDQyVLMSSPVGR	IRS2_HUMAN	9.894209036	9.894209036		
ASSPAESSPEDSGyMR	IRS2_HUMAN	3.579110975	2.661095047		
SSSSNLGADDGyMPMTPGAALAGSGSGScR	IRS2_HUMAN	8.691303295	0.557579673		
LEyYESEKK	IRS2_HUMAN	-7.888224473	-8.385299688	0	-5.943703854
LEyYESEK	IRS2_HUMAN	1.059366066	0.993633795		
GPGAGGDEATAGGGSAPQPPRLEYyESEK-25	IRS2_HUMAN	0	-5.413701014		
GPGAGGDEATAGGGSAPQPPRLEyYESEKK	IRS2_HUMAN	0	-7.862527175		
GPGAGGDEATAGGGSAPQPPRLEyYESEK-24	IRS2_HUMAN	-6.208461659	-5.555353642		
WDTGENPIyK	ITB1_HUMAN	0.610040922	0.457293137		
WDTAnNPLyK	ITB3_HUMAN	0.610040922	0.457293137		
VcAYGAQGEGPySSLVScR	ITB4_HUMAN	-8.160703694	-8.400523094		
LIyLVPEK	ITSN2_HUMAN			7.020766435	7.020766435
REEPEALyAAVNK	ITSN2_HUMAN	1.013569467	0.955649209		
REVGDyGQLHETEVLLK	JAK2_HUMAN	0	-7.730098493		
VIySQPSAR	JAM1_HUMAN	-0.46722275	-0.869557692		
KVIySQPSAR	JAM1_HUMAN	0	-7.182285023		
SLyNLGGSKR	K2C5_HUMAN				
SAyGGPVGAGIR	K2C7_HUMAN				
LSSARPGGLGSSSLyGLGASRPR	K2C7_HUMAN	-8.077551503	-7.94667673	0.185688831	-0.135071823
DVDAAyMSK	K2C7_HUMAN	0	-2.891454124		
FSYTSQHSDyGQR	KIRR1_HUMAN	1.510873731	7.366662259		
EEYEMKDPTNGyYNVR	KIRR1_HUMAN	0.597208282	1.310916783		
AVLyADYR	KIRR1_HUMAN	-0.228098013	9.415582766		
LSHSSGyAQLNTYSR-7	KIRR1_HUMAN	-0.360231937	0.238783904		

AIySSFKDDVDLK	KIRR1_HUMAN	-1.774789119	-1.04165094		
LSHSSGYAQLNTySR-13	KIRR1_HUMAN	-3.260695999	-4.348769255		
FNSHPFPGAAGyPTYR	KIRR1_HUMAN	-3.544044542	0		
RSDSASSEPVGIyQGFEK	KPCD_HUMAN	10.49528707	2.295330359		
SDSASSEPVGIyQGFEK	KPCD_HUMAN	1.742678585	1.008393891		
KTGVAGEDMQDNSGTyGK	KPCD_HUMAN	5.189808903	5.189808903		
TGVAGEDMQDNSGTyGK	KPCD_HUMAN	1.771081964	1.446551244		
TATESFASDPILyRPVAVALDTK	KPYM_HUMAN			-0.127932054	-0.439535098
EAEAAIyHLQLFEELRR	KPYM_HUMAN	0	-6.621553129		
AQIPEGDyLSYR	LAP2_HUMAN				
IyDILSDNGPQQPSTTVK	LAP2_HUMAN	-0.403558231	1.435884797		
NLDNGGFyISPR	LCK_HUMAN			0.667923373	-0.355526989
QVVESAyEVIK	LDHA_HUMAN	1.165489139	0.421259866	0.196431165	0.092281076
DQLIyNLLKEEQTPQNK	LDHA_HUMAN	-6.851497563	-6.338647312		
MVVESAyEVIK	LDHB_HUMAN	-0.754883629	-0.132776684	0.105457139	-0.151182293
TTEDEVHIcHNQDGySYPSR-15	LDLR_HUMAN	-0.576545335	0.228029944		
TTEDEVHIcHNQDGYSyPSR-17	LDLR_HUMAN	-7.637838716	0		
NmDDyEDFDEK	LMBD2_HUMAN	5.586726024	5.586726024		
NHQLYcNDcyLR	LMO7_HUMAN	-7.369380455	-7.592317357		
NDSDPTyGQQGHPNTWK	LPP_HUMAN	1.134263855	0.948585713		
SAQPSPHYMAAPSSGQIyGSGPQGYNTQPVPVSGQcPPPSTR-18	LPP_HUMAN	-3.495198532	-3.203246709		
SAQPSPHYMAAPSSGQIYGSGPQGyNTQPVPVSGQcPPPSTR-25	LPP_HUMAN	-5.144623959	0		
SAQPSPHyMAAPSSGQIYGSGPQGYNTQPVPVSGQcPPPSTR-8	LPP_HUMAN	-7.655067005	0		
SSSAGGQGSyVPLLR	LSR_HUMAN	-8.00946321	-7.473528357		
VIEDNEyTAR	LYN_HUMAN	0.598716375	0.611509459	1.918734287	1.691727049
SLDNGGyYISPR-7	LYN_HUMAN	0.284516502	-0.087485777		
SLDNGGYyISPR-8	LYN_HUMAN	0.284516502	-0.087485777		
VENcPDELyDIMK	LYN_HUMAN	0.458406263	-2.094778446		
TPDTSTYcyETAEK	MAP1B_HUMAN	0	-5.048218557		
AAEAGGAEEQyGFLTTPTK	MAP1B_HUMAN	-1.532436535	-1.326574993		
TSDVGGYYyEK-9	MAP1B_HUMAN	-4.967757011	-5.923492342		
TSDVGGyYYEK-7	MAP1B_HUMAN	-4.967757011	-5.923492342		
TSDVGGYyYEK-8	MAP1B_HUMAN	-4.967757011	-5.923492342		
DMYDKEyYSVHNK-7	MET_HUMAN	-0.50125845	10.72270418		
DmyDKEYYSVHNK-3	MET_HUMAN	-8.343068487	0		
yWKPTGTPK	MGLL_HUMAN	7.231121917	2.033924394		
VADPDHDHTGFLTEyVATR	MK01_HUMAN	2.430534071	1.749655225	4.40303971	3.589873135
IADPEHDHTGFLTEyVATR	MK03_HUMAN	2.041210957	1.890153039	9.400194047	9.400194047
GLcTSPAEHQYFMTEyVATR	MK07_HUMAN	0	-8.700623137		
TAGTSFMMTPyVVTR	MK08_HUMAN	0	-7.852881309		
TAcTNFMMTPyVVTR	MK09_HUMAN	0.86282809	2.242665225	0	-8.565402751
QADSEMTGyVVTR	MK12_HUMAN	0.428281197	1.46843141		
HADAEmTGyVVTR	MK13_HUMAN				
HTDDEMTGyVATR	MK14_HUMAN	1.004587636	0.82936758	8.102830484	8.102830484
SLPSGSHQGPVIyAQLDHSGGHHSDK	MPZL1_HUMAN	1.509407288	0.14672114	12.03053111	12.03053111

SESVVyADIR	MPZL1_HUMAN	-0.481359099	-1.151243131		
DTYHPMSEYPTyHTHGR-12	MUC1_HUMAN	1.153726553	1.486637966		
YVPPSSTDRSPyEK	MUC1_HUMAN	0.742076928	1.021428994		
DTYHPMSEyPTYHTHGR-9	MUC1_HUMAN	0.651746547	1.259387608		
DTyHPMSEYPTYHTHGR-3	MUC1_HUMAN	0.397294142	1.154745974		
VSAGNGGSSLSyTNPAVAATSANL	MUC1_HUMAN	0	-7.68222088		
IPPyHYIHVLDQNSNVSR-4	MVP_HUMAN			0.372185184	-0.20999021
IPPYHyIHVLDQNSNVSR-6	MVP_HUMAN			8.141349509	-0.20999021
VAAyDKLEK	MYH9_HUMAN	0	-6.936553086	6.724376388	6.724376388
YLLETSGNLDGLEyK	NAMPT_HUMAN	0	-4.790001324		
LyDLNMPAYVK	NCK1_HUMAN	1.942341709	1.271149529	7.530124087	7.530124087
RKPSVPDSASPADDSFVDPGERLyDLNMPAYVK	NCK1_HUMAN	0	-7.551247332		
RPLHPALNQPGGLQPLSFQNPVyHLNNPIPAMPK	NGAP_HUMAN	1.491866419	1.093434585		
TSFNyAMK	NQO1_HUMAN	0	-8.024281361		
DKLNTQSTySEA	NRP1_HUMAN	-0.079303401	0.239076791	0	-5.733492762
TLHyEcIVLVK	NUDT5_HUMAN			-0.170586756	-0.489071579
FNqKGEVyK	ODB2_HUMAN	0	-11.55291992		
LyEEYTR	P55G_HUMAN	0	-3.898123156		
SKEYDRLyEEYTR	P55G_HUMAN	0	-7.057120664		
TRDQyLMWLTQK	P85A_HUMAN	-6.626650716	-3.730019511		
SREYDQLyEEYTR	P85B_HUMAN	0.91403529	0.091398626		
EYDQLyEEYTR	P85B_HUMAN	0.695755985	0.000654284		
NETEDQyALMEDEDDLPHHEER	P85B_HUMAN	-9.674428573	-8.199596712		
SPSScNDLyATVK	PAG1_HUMAN	7.673074298	0.480394147		
ENDyESISDLQQGR	PAG1_HUMAN	0.977721277	0.703345513		
SGQSLTVPESTyTSIQGDPQR	PAG1_HUMAN	-5.800007482	-7.779984769		
SVDGDQGLGMEGPyEVLK	PAG1_HUMAN	-7.55539386	-6.966181188		
SREEDPTLTEEEISAMySSVNKPGQLVNK	PAG1_HUMAN	-8.776614787	-6.356215689		
SSFSHySGLK	PAIRB_HUMAN	-6.752149337	-6.127421646		
VGEEEHVySFPNK	PAXI_HUMAN	0.496548202	0.381259869	-0.122902145	-0.377915758
FIHQQPQSSSPVyGSSAK	PAXI_HUMAN	0.18533264	0.425759213	0.161108554	-0.023018217
LDnmLLAEGVSGPEKGGGSAAAAAAAASGGSSDNSIEHSDyR	PBX3_HUMAN	0	-6.388731377		
EPSAPSIPTPAyQSSPAGGHAPTPPTPAPR	PDC6I_HUMAN	-9.771595092	-9.512553667	-7.863399641	-8.724385376
ALNGAEPNyHSLPSAR	PDRO_HUMAN	-6.927303227	-7.393293938		
VPIVINPNAyDNLAIYK-10	PEAK1_HUMAN	-1.516454162	-1.290756591	0.072160629	7.262985714
VPIVINPNAYDNLAIyK-16	PEAK1_HUMAN			0.072160629	7.262985714
ASTDVAGQAVTINLVPTEEQAKPyR	PEAK1_HUMAN	8.478160819	8.478160819		
EDGKEDISDPMDPNPcSATySNLGQSR	PEAK1_HUMAN	-8.249567491	-7.970674312		
ATFHTPFSHLGQSPEGcSSyTFPK	PECI_HUMAN	-0.013146382	0.189969953		
FSGWyDADLSPAGHEEAKR	PGAM1_HUMAN				
HyGGLTGLNK	PGAM1_HUMAN	0	-8.900172419		
LSPGESAyQK	PHTNS_HUMAN	7.498491268	0.082314288		
FKEycPMVFR	PI42A_HUMAN	0	-7.843887672		
FGyHIIMVEGR	PIN4_HUMAN	0	-6.952727071		
SEDIyADPAAYVmR	PKHA6_HUMAN	-4.421489361	-6.879030905		

AGTTATyEGR	PKP2_HUMAN				
NLIyDNADNK	PKP3_HUMAN				
NNYALNTTATyAEPYRPIQYR	PKP4_HUMAN	-8.810084226	0		
LNyGIPAIVK	PLAK_HUMAN			1.110827613	-0.191513289
AVTGYKDPySGK	PLEC_HUMAN			5.488867387	5.488867387
GYYSPySVSGSGSTAGSR	PLEC_HUMAN	0.412623785	-0.102939559		
cccIyEKPR	PP1RB_HUMAN	0.073791965	0.083238892		
cyEMASHLR	PROF1_HUMAN				
LcDFGSASHVADNDITPyLVSR	PRP4B_HUMAN	0.546362243	-0.726694879	-0.162394658	-0.754806341
HIGLVySGMGPDYR	PSA2_HUMAN	-6.325107155	-5.773752222		
KGPGLyYVDEHGTR	PSB8_HUMAN	0	-6.99601711		
GQPIyIQFSNHK	PTBP1_HUMAN	-7.75891605	-8.360304137		
IQNTGDyYDLYGGEK-7	PTN11_HUMAN	1.171353714	0.228770298	8.039702623	8.039702623
IQNTGDYyDLYGGEK-8	PTN11_HUMAN	1.171353714	0.228770298	8.039702623	8.039702623
IQNTGDYYDLyGGEK-11	PTN11_HUMAN			8.039702623	8.039702623
VyENVGLMQQQK	PTN11_HUMAN	3.116138233	2.300745902		
GQESEyGNITYPPAMK	PTN6_HUMAN	-5.655349056	-3.563124374		
VVQEYIDAFSDyANFK	PTPRA_HUMAN	0.727976752	-0.735594345	-0.555012474	7.464228205
VVQDFIDIFSDyANFK	PTPRE_HUMAN			5.964392134	5.964392134
VMIyQDEVK	PTRF_HUMAN				
SFTPDHVVyAR	PTRF_HUMAN	1.214211117	1.06806252		
VMIyQDEVKLPAK	PTRF_HUMAN				
KSFTPDHVVyAR	PTRF_HUMAN	-9.867120425	0		
YGLFKEENPyAR	PTTG_HUMAN	-7.608227865	0		
VVAcNLyPFVK	PUR9_HUMAN	-7.335788102	-7.758722886	-0.19581232	-0.723768469
ARPEyMLPVHFYGR	PYGB_HUMAN			0	-8.524694344
AHAWPSPyKDYEVK	RAI3_HUMAN			9.490311539	9.490311539
AYSQEEITqGFEETGDTLyAPYSTHFQLQNQPPQK-19	RAI3_HUMAN			5.942634725	5.942634725
AYSQEEITQGFEETGDTLYAPySTHFQLQnQPPQK-22	RAI3_HUMAN			5.942634725	5.942634725
AHAWPSPyKDyEVKK	RAI3_HUMAN			11.30122361	0.853996041
NLQyYDISAK-4	RAN_HUMAN			0	-8.630639379
NLQYyDISAK-5	RAN_HUMAN	0	-6.794337921	0.043525784	-0.188468513
SNyNFEKPFLWLAR	RAN_HUMAN			1.815541475	1.309854036
HFTNPycNIYLNSVQVAK	RASA1_HUMAN	0.15831618	-0.144128687	0.214445569	-0.474229323
ASyVAPLTAQPATYR	RBM14_HUMAN				
MQAYSNPGySSFPSPTGLEPScK	RFFL_HUMAN	-7.290129436	-7.451653591		
EKPAQDPLyDVPNASGGQAGGPQRPGR	RIN1_HUMAN	0.825513171	1.64542798	0.706403157	0.134376668
IIQLLDDyPK	RLA0_HUMAN	0.202540112	-0.052520716	0.0358663	-0.621892047
AVPKEDIySGGGGGGSR	ROA0_HUMAN	0.892558512	0.697164042		
EDIySGGGGGGSR	ROA0_HUMAN	0.386315747	0.552091863		
SSGPYGGGGQyFAKPR-11	ROA1_HUMAN	0.450281446	0.40233303		
SSGPyGGGGQYFAKPR-5	ROA1_HUMAN	1.691655161	1.072436008		
NQGGYGGSSSSSSyGSGR-14	ROA1_HUMAN	-0.183303647	0.175250818		
NQGGyGGSSSSSSYGSGR-5	ROA1_HUMAN	-0.63727579	-0.542500974		
NMGGPyGGGNYGPGGSGGSGGYGGR-6	ROA2_HUMAN	0.936037546	0.261464111		

NMGGPYGGGNYGPGGSGGSGGyGGR-22	ROA2_HUMAN	0.605281606	0.717412902		
NMGGPYGGGNyGPGGSGGSGGYGGR-11	ROA2_HUMAN	-0.367758899	-0.482774335		
SSGSPyGGGYGSGGGSGGYGSR-6	ROA3_HUMAN	1.406058444	-0.038254307		
SSGSPYGGGYGSGGGSGGyGSR-19	ROA3_HUMAN	0.421720549	0.171932147		
SSGSPYGGGyGSGGGSGGYGSR-10	ROA3_HUMAN	0	-6.633582564		
NGLTSTyAGIR	ROBO1_HUMAN	0	-4.846393569		
IAIyELLFK	RS10_HUMAN	-6.314542848	0	-8.483237003	-8.738154146
LVQSPNSyFMDVK	RS27_HUMAN	0.276863866	0.424545869	7.018628628	-0.31817759
ADHQPLTEASyVNLPTIALcNTDSPLR	RSSA_HUMAN	-5.749005678	-6.857360973		
EFEVyGPIKR	RU17_HUMAN	6.315387916	-2.055867168		
FAGDKGyLTK	S10AA_HUMAN	-4.613658355	-4.613577602		
SHyADVDPENQNFLLESNLGK	S38A2_HUMAN				
QmAyENLnDK	SCG2_HUMAN	5.097935066	5.097935066		
VIQAQTAFSANPANPAILSEASAPIPHDGNLyPR	SDCB1_HUMAN	-6.116242182	-6.443601074	-0.038445329	-0.26131802
QQPTQFINPETPGyVGFANLPNQVHR	SEPT2_HUMAN	0.485555864	0.464100016		
NQDATVyVGGLDEK	SF3B4_HUMAN	0	-6.692399779		
EATQPEPIyAESTK	SG223_HUMAN	0.179465641	0.173217608	-0.069258643	-0.253038703
EATQPEPIyAESTKR	SG223_HUMAN	10.04306915	0.275634177	-0.273611036	-0.381303313
AGKGESAGyMEPYEAQR	SHB_HUMAN	3.958040214	3.958040214		
GESAGyMEPYEAQR	SHB_HUMAN	0.125348887	2.151883669		
VTIADDySDPFDAK	SHB_HUMAN	-6.969063912	-5.368815852		
DKVTIADDySDPFDAK	SHB_HUMAN	-7.366968878	-5.793757121		
ELFDDPSyVNVQNLDK	SHC1_HUMAN	1.427467166	1.546842182	1.841576929	1.801339667
MAGFDGSAWDEEEEEPPDHQYyNDFPGK	SHC1_HUMAN	0	-5.649729871		
APPcSGSSITEIINPNyMGVGPFGPPMPLHVK	SHIP1_HUMAN	3.986018279	3.986018279		
NSFNNPAyYVLEGVPHQLLPPEPPSPAR-8	SHIP2_HUMAN	5.677892677	7.780134346	4.361323377	0.985713003
NSFNNPAYyVLEGVPHQLLPPEPPSPAR-9	SHIP2_HUMAN			4.361323377	0.985713003
TLSEVDyAPAGPAR	SHIP2_HUMAN	7.269453674	7.269453674		
LGQDPyR	SON_HUMAN	0	-4.165679466		
EDLELYyKK	SPAT1_HUMAN	-9.862304965	0		
ALSSEGKPyVTKEELYQNLTR	SPTA2_HUMAN	0	-5.959573616		
cSVcPDyDLcSVcEGK	SQSTM_HUMAN	2.402568125	1.930439378		
LPSSPVyEDAASFK	SRC8_HUMAN	-0.394169872	0.324421464	1.201578705	8.385094417
TQTPPVSPAPQPTEERLPSSPVyEDAASFK	SRC8_HUMAN	-1.355332174	-1.082415498		
GPVSGTEPEPVySMEAADYR	SRC8_HUMAN	-1.966442085	-0.899721807		
SHEGETAyIR	SRSF1_HUMAN	1.575511779	6.396591187	0	-5.476605395
YcRPESQEHPEADPGSAAPyLK	STAT3_HUMAN	3.503882973	2.950444426	0.985061505	1.008133972
GASQAGMTGyGMPR	TAGL2_HUMAN	-0.525203724	-0.33961027		
IHFPLATyAPVISAEK	TBA1A_HUMAN;TBA1B_HUMAN;TBA1C_H	-0.408782724	-0.780699178		
VGINyQPPTVVPGGDLAK	TBA1A_HUMAN;TBA1B_HUMAN;TBA1C_H	-6.052781629	-6.877886617		
QLFHPEQLITGKEDAANNyAR	TBA1C_HUMAN;TBA4A_HUMAN				
LGEyEDVSR	TBCB_HUMAN	1.184576706	1.49226466		
LSLGQyDNDAGGQLPFSK	TENS3_HUMAN	1.38360526	0.598153685	0.152384565	0.111447588
ESMcSTPAFPVSPETPyVK	TENS3_HUMAN	1.231516023	0.399095589	-6.986569823	0
KLSLGQyDNDAGGQLPFSK	TENS3_HUMAN	9.803447521	0.390945363	0.246000816	0.595372195

QQQMVVAHQySFAPDGEAR	TENS3_HUMAN	0.753080021	0.466244696		
SAFSNLFGGEPLSyTR	TFR1_HUMAN	-6.266613951	0		
SEGTyccGPVPVR	TGM2_HUMAN	0	-5.728206073		
MIMDTAGNDPycFVEFHEHR	TIA1_HUMAN	0	-7.077301356		
MITEHTSNDPycFVEFYEHR	TIAR_HUMAN	0	-7.000419715		
yyGAVGSTLR	TITIN_HUMAN				
NmAEQIIQEIySQIQSK	TKT_HUMAN	0	-4.214159594		
ALDyYMLR-4	TLN1_HUMAN	-6.200741821	-5.142970014	0.028178365	-0.314322633
TMQFEPSTMVyDAcR	TLN1_HUMAN	0.322350495	1.939952185	-0.324793126	-0.655566335
ALDYyMLR-5	TLN1_HUMAN			0.028178365	-0.314322633
AVSSAIAQLLGEVAQGNENyAGIAAR	TLN1_HUMAN			4.377372038	4.377372038
KSTVLQQQyNR	TLN1_HUMAN	6.223612058	-0.105145379		
STVLQQQyNR	TLN1_HUMAN	0	-3.135745449		
AKPEPDILEEEKIyAYPSNITSETGFR	TM192_HUMAN	0	-3.984543781		
KVAWDyGR	TM63B_HUMAN	-5.806975601	0		
KVVYENAyGQFIGPHR	TRXR1_HUMAN	0.494948361	0.429798592		
VVYENAyGQFIGPHR	TRXR1_HUMAN	0.3028796	0.150476847		
SyDYDLIIIGGGSGGLAAAK	TRXR1_HUMAN	-6.254788061	-4.586902353		
SYDyDLIIIGGGSGGLAAAK	TRXR1_HUMAN	-6.254788061	-4.586902353		
RTDAySR	TSC2_HUMAN	0	-5.067493385		
LLAQAEGEPcyIR	TYK2_HUMAN	0.427550144	0.650290686	0.271371896	-0.179704057
NSQEAEVScPFIDNTyScSGK	UB7I3_HUMAN	0	-4.653806419		
IYNGDyYR-6	UFO_HUMAN	-6.571866161	-4.028400689		
IYNGDYyR-7	UFO_HUMAN				
KIYNGDYyR-8	UFO_HUMAN	-5.087437116	0		
KIYNGDyYR-7	UFO_HUMAN	-5.087437116	-4.202602173		
ESSSIyISK	UGDH_HUMAN	0.087180155	-1.387041169		
IEQyATR	UTRO_HUMAN	0	-4.84524289		
VQIyHNPTANSFR	VASP_HUMAN	7.633845494	0.331333511	0.103225577	-0.164479678
MDyVEINIDHK	VIGLN_HUMAN	-9.083715404	-8.142259027		
SLYASSPGGVyATR-11	VIME_HUMAN	1.172722507	0.939840216	0.879416418	8.930785673
SLyASSPGGVYATR-3	VIME_HUMAN	0.951824117	0.696272775		
SyVTTSTR	VIME_HUMAN	0	-4.013020641		
TySLGSALRPSTSR	VIME_HUMAN	0	-7.273970914		
FANyIDKVR	VIME_HUMAN	0	-7.393134987		
FANyIDK	VIME_HUMAN	-6.460876689	-5.317123028		
SFLDSGyR	VINC_HUMAN	0.006567089	-0.001754761	-0.134164237	-0.404154908
VIyDFIEK	WASL_HUMAN	-10.13478753	-10.48207747	0.393351676	0.551334714
AHDGGIyAISWSPDSTHLLSASGDK	WDR1_HUMAN	-8.866399127	-9.132921455	-0.107690085	-0.606930955
AGEEHyNcISALHK	WRIP1_HUMAN	0	-6.910635107		
DHVVEGEPyAGYDR	XYLK_HUMAN	0	-6.689527226		
GESyNcGYKPYR	ZFHX2_HUMAN				
QTPSLPEPKPVyAQVGQPDVDLPVSPSDGVLPNSTHEDGILRPSm	ZO1_HUMAN	0	-2.323324016		
HEEQPAPGyDTHGR	ZO1_HUMAN	-5.51834817	0		
